# Slow induction of photosynthesis on shade to sun transitions in wheat may cost at least 21% of productivity

**DOI:** 10.1098/rstb.2016.0543

**Published:** 2017-08-14

**Authors:** Samuel H. Taylor, Stephen P. Long

**Affiliations:** 1 Lancaster Environment Centre, Lancaster University, Lancaster, Lancashire LA1 4YQ, UK; 2 Department of Crop Sciences, University of Illinois, Urbana, IL 61801, USA; 3 Department of Plant Biology, University of Illinois, Urbana, IL 61801, USA

**Keywords:** food security, Rubisco, Rubisco activase, photosynthetic induction, wheat, crop yield improvement

## Abstract

Wheat is the second most important direct source of food calories in the world. After considerable improvement during the Green Revolution, increase in genetic yield potential appears to have stalled. Improvement of photosynthetic efficiency now appears a major opportunity in addressing the sustainable yield increases needed to meet future food demand. Effort, however, has focused on increasing efficiency under steady-state conditions. In the field, the light environment at the level of individual leaves is constantly changing. The speed of adjustment of photosynthetic efficiency can have a profound effect on crop carbon gain and yield. Flag leaves of wheat are the major photosynthetic organs supplying the grain of wheat, and will be intermittently shaded throughout a typical day. Here, the speed of adjustment to a shade to sun transition in these leaves was analysed. On transfer to sun conditions, the leaf required about 15 min to regain maximum photosynthetic efficiency. *In vivo* analysis based on the responses of leaf CO_2_ assimilation (*A*) to intercellular CO_2_ concentration (*c*_i_) implied that the major limitation throughout this induction was activation of the primary carboxylase of C3 photosynthesis, ribulose-1,5-bisphosphate carboxylase/oxygenase (Rubisco). This was followed in importance by stomata, which accounted for about 20% of the limitation. Except during the first few seconds, photosynthetic electron transport and regeneration of the CO_2_ acceptor molecule, ribulose-1,5-bisphosphate (RubP), did not affect the speed of induction. The measured kinetics of Rubisco activation in the sun and de-activation in the shade were predicted from the measurements. These were combined with a canopy ray tracing model that predicted intermittent shading of flag leaves over the course of a June day. This indicated that the slow adjustment in shade to sun transitions could cost 21% of potential assimilation.

This article is part of the themed issue ‘Enhancing photosynthesis in crop plants: targets for improvement’.

## 1. Introduction

Leaves of crops in the field experience frequent fluctuations in light, moving from shade to full sunlight, and vice versa, as clouds obscure the sun or as leaves go into the shade of other leaves, stems and floral structures. Recently, it was shown that increasing the rate at which leaves could re-adjust photosynthetic efficiency on transfer to shade increased productivity of a tobacco crop in replicated field trials by 14–20% [1]. This was shown to result from a decrease in the time required for non-photochemical quenching to relax and the efficiency of leaf photosynthetic CO_2_ uptake (*A*), in limiting light, to recover. Equally, there is a lag in achieving maximum efficiency when leaves are transferred in the opposite direction from shade to sun. The increase in *A* that occurs following the transition has been termed photosynthetic induction [2]. Although many factors could govern the speed of induction, it has been shown to correlate with, and modelled to correspond to, Rubisco activation in, for example, soya bean and tobacco [3,4]. More recently, over-expression of *Rca*, the gene coding for Rubisco activase (Rca), in rice resulted in a slightly increased speed of induction at 25°C [5]. *In vivo*, the steady-state response of leaf CO_2_ uptake (*A*) to intercellular CO_2_ concentration (*c*_i_) has proved a highly valuable means to partition limitations, including apparent Rubisco activity (*V_c_*_,max_). Recently, this concept has been extended by inducing photosynthesis on the same leaf in a range of CO_2_ concentrations. This allowed the production of dynamic *A/c*_i_ responses to infer limitations at different stages of induction in soya bean. On transfer of leaves from a shade light level of 100 µmol m^−2^ s^−1^ to a full sun level of 2000 µmol m^−2^ s^−1^, 10–20 min were required for leaves to regain full efficiency. The dynamic *A/c*_i_ analysis over this period inferred that the slowest responding determinant of photosynthetic rate was Rubisco activity, suggesting activation of this enzyme as the primary cause of this delay [3,4]. However, the impact this might have on production was not quantified.

Bread wheat (*Triticum aestivum* L.) is second only to rice in importance to the world's population as a direct source of food calories [6]. After large improvements in global yields of wheat per hectare following the Green Revolution, improvement stagnated in the first decade of this century [6–9]. Improved partitioning of biomass to grain, i.e. harvest index, was roughly doubled, making it the key factor of genetic improvement of yield potential during the Green Revolution. Harvest index is now at about 60% of total shoot biomass in contemporary cultivars, and is close to its biological limits [10,11]. This may explain why increases in yield potential have been stagnating in recent years. New innovations are therefore needed if genetic yield potential of wheat is to be improved further [10,12]. Photosynthetic efficiency in wheat, as in all crops, falls well short of its theoretical potential and has been improved little with selection and breeding [13]. Indeed some have argued that leaf photosynthetic capacity has decreased with domestication [14].

The flag leaf of wheat, together with the ear, are considered to account for most of the carbohydrate that accumulates in the developing grain [15]. Furthermore, the proportion of photosynthate derived from the flag leaf relative to the ear has increased progressively with the increase in harvest index through the past 50 years [16], so increasing its importance as a source of carbohydrate for the developing grain. Using a current cultivar of wheat, this study: (i) determines the speed of adjustment of photosynthesis in the flag leaf on transfer from shade to sun; (ii) infers, by developing dynamic *A/c*_i_ responses, the *in vivo* factors determining the speed of adjustment; and (iii) estimates the loss of potential production that may result from this slow adjustment.

## 2. Material and methods

### (a) Plant material and growth conditions

A bread-making quality wheat (*Triticum aestivum* L.) cv. Highbury was used (Nottingham University, UK). Seed was sown into 3 l containers of soil-less compost mix (Petersfield Products, Leicester, UK) incorporating a broad range fertilizer (PG Mix, Yara, Grimsby, UK), in a controlled environment greenhouse. Day/night temperatures were maintained at 24 ± 9.3°C/19 ± 1.4°C (mean ± s.d.) and relative humidity was 45 ± 12.6%. Growth CO_2_ concentration in the greenhouse air was measured hourly and averaged 449 ± 23 µmol mol^−1^ over the duration of the experiment. Daylight was supplemented with high pressure sodium lamps (SON-T 400 W, Philips Lighting, Eindhoven, The Netherlands) to ensure a minimum photosynthetic photon flux density (PPFD) of 500 µmol m^−2^ s^−1^ at the plant surface for 16 h d^–1^. After germination, seedlings were thinned to one per container. Containers were watered daily to field capacity.

### (b) Gas exchange and analysis of photosynthetic CO_2_ responses

Photosynthetic gas exchange of fully emerged flag leaves was measured between heading and anthesis. The mid-section of the leaf was enclosed within a controlled environment cuvette integrated into a portable gas exchange system incorporating infrared CO_2_ and water vapour analysers (LI-6800F, LI-COR, Lincoln, NE). Light was provided through the light-emitting diodes incorporated into the cuvette head.

Response curves of net leaf CO_2_ uptake (*A*) to PPFD were determined to obtain preliminary values for day respiration (*R*_d_) and identify the lowest PPFD that would be saturating for subsequent static and dynamic *A/c*_i_ analysis. In all measurements, leaf temperature was maintained at 25°C and leaf vapour pressure deficit (VPD_leaf_) at *ca* 1.0 kPa. Transpiration was measured simultaneously to determine stomatal conductance to water (*g*_s,w_), to correct for impacts on measured CO_2_ fluxes, and to allow calculation of *c*_i_ based on transpiration-corrected leaf conductance to CO_2_. Leaves were induced to steady state at a cuvette CO_2_ of 400 µmol mol^−1^ and a PPFD of 1500 µmol m^−2^ s^−1^, allowing at least 40 min for steady state to be achieved. PPFD was then stepped down through 1200, 1000, 800, 600, 500, 400, 300, 200, 150, 100, 50 and 0 µmol m^−2^ s^−1^; measurements were collected immediately cuvette conditions stabilized at each light level. The response of *A* to incident PPFD was then fit using nonlinear least squares (*nls*: R Language and Environment) to a non-rectangular hyperbola [17]:

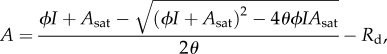
where *ϕ* is the realized quantum yield (mol mol^−1^); *I*, incident PPFD (μmol m^−2^ s^−1^); *A*_sat_, the maximum gross rate of leaf CO_2_ assimilation (μmol m^−2^ s^−1^); *θ*, a dimensionless curvature parameter; *R*_d_, the daytime rate of respiration (μmol m^−2^ s^−1^). Fitted values were (mean ± s.e.): *ϕ*, 0.067 ± 0.0049; *A*_sat_, 38.1 ± 3.58; *θ*, 0.58 ± 0.044; *R*_d_, 1.68 ± 075; *R*_d_ was used as an initial value in models of the photosynthetic response to CO_2_ concentration.

The ‘static’ response of *A* to *c*_i_ (expressed as the mole fraction in air: μmol mol^−1^) was determined by obtaining steady-state *A* under the conditions described above, but by maintaining PPFD at 1200 µmol m^−2^ s^−1^ and varying CO_2_ in the air surrounding the leaf (*c*_a_). Measurements were made at 430, 300, 200, 150, 100, 50 and approximately 0 µmol mol^−1^ *c*_a_, which was then increased to 430, 500, 600, 800 and 1000 µmol mol^−1^; following procedures recommended previously [18]. Values for *A* and *c*_i_ were calculated from the equations of Farquhar & von Caemmerer [19].

Parameters of the response of *A* to *c*_i_ were characterized on the basis of limitation by Rubisco (*A*_C_) and electron transport (*A*_J_) [19].

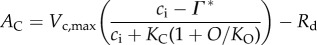
and

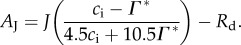


The maximum rate of carboxylation (*V_c_*_,max_, μmol m^−2^ s^−1^), the rate of electron transport (*J*, μmol m^−2^ s^−1^), and *R*_d_ were fit using nonlinear least squares. To do this, values for: Γ*, the photorespiratory compensation point; *K*_C_, the Rubisco Michaelis constant for CO_2_; and *K*_O_, the Rubisco Michaelis constant for O_2_, were calculated at the mean leaf temperature, based on values for tobacco following Bernacchi *et al*. [20]. Using nonlinear least squares, *V_c_*_,max_ and *R*_d_ were estimated first, and the value of *R*_d_ was used when estimating *J*. Parameters were normalized to 25°C following previously described relationships to temperature [20]. Because calculation of the true *V*_c__,max_ requires determination of *c*_c_, we note that the term determined here from *c*_i_ and referred to as *V_c_*_,max_ is determined by both the *in vivo* activity of Rubisco and mesophyll conductance (*g*_m_).

To identify the transition point between Rubisco and ribulose-1,5-bisphosphate (RuBP) limitation, we used an approach derived from the recommendations of Gu *et al*. [21]. All possible combinations of *A*_C_ and *A*_J_ were fit to each CO_2_ response curve, and the best fit was selected based on the minimal value of 

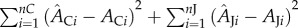
where 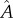 are predicted, and *A* observed values for the respective segments of the *A*/*c*_i_ curves. The best fitting *A*_C_, *A*_J_ combination was considered admissible if the transition point predicted fell between data assigned to *A*_C_ and *A*_J_. Stomatal limitation (*l*) was also calculated from the *A*/*c_i_* response [22],

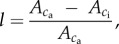
where 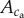 is the value of *A* as determined from the *A*/*c*_i_ response if *c*_i_ = *c*_a_, i.e. assuming infinite boundary layer and stomatal conductances. 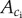 is the actual *A* achieved at the given *c*_a_, i.e. accounting for the decrease in *c*_i_ resulting from the actual stomatal conductance (*g*_s_).

To determine the limitations to *A* during low to high light transitions leaf gas exchange was measured at a range of *c*_a_ and ‘dynamic’ *A/c*_i_ responses constructed as described previously [24]. At the start of measurements each leaf was brought to steady state at a *c*_a_ of 400 µmol mol^−1^, PPFD of 1200 µmol m^−2^ s^−1^, cuvette air temperature of 25°C, and VPD *ca* 1.0 kPa. Induction measurements followed decreases in PPFD to 50 µmol m^−2^ s^−1^ for 30 min (shade): gas exchange was recorded at 10 s intervals for 15 min following a step change back to ‘sun’ (1200 µmol m^−2^ s^−1^ PPFD), a PPFD sufficient for saturation of *V_c_*_,max_ and *J*. The cycle of 30 min shade + 10 min sun was repeated at *c*_a_ of 50, 100, 200, 300, 400, 500, 600, 800 and 1000 µmol mol^−1^. Within a few seconds after the transition from ‘shade’ to ‘sun’, leaf temperatures rose by approximately 1°C to the range 24.5–25.1°C, with coefficients of variation (CV) during inductions less than 0.63%. The range of leaf VPD during inductions was 1.0–1.2 kPa, with CV < 2.8%; CV for *c*_a_ were less than 3%. In the shade at ambient and higher cuvette *c*_a_, *g*_s_ decreased, minimizing the range of *c*_i_ that could be obtained and preventing characterization of *A*_J_. To fully characterize photosynthetic limitations during induction dynamic *A*/*c*_i_ measurements were repeated, but using a *c*_a_ of 100 µmol mol^−1^ during shade to inhibit stomatal closure before switching to the desired *c*_a_ and sun condition for induction.

CO_2_ response curves were fit to the data for each 10 s interval of induction. A small number of inadmissible fits were obtained when there was insufficient data to fit both *A*_C_ and *A*_J_; we re-fit these cases using either *A*_C_ or *A*_J_ (alongside *R*_d_), and chose the best fit based on a comparison of 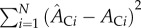 and 
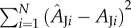
. To determine whether *A* during photosynthetic induction was limited primarily by *V_c_*_,max_ or *J*, parameters from the dynamic *A*/*c*_i_ responses were used in combination with steady-state *g*_s,w_ to estimate a maximum probable operating *c*_i_: 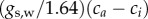 equated to 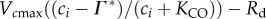. The resulting quadratic was solved for *c*_i_ at each 10 s interval through induction.

### (c) *In vivo* kinetics for Rubisco activation in wheat

The time constant for Rubisco activation was determined from the kinetics of *A* following transitions from low to high light, excluding transient changes occurring during the first minute as described previously [23]

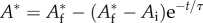


where 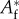 is a steady-state value for A*: the potential gross leaf CO_2_ assimilation in sun, corrected to constant *c*_i_. A* was calculated as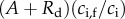, where *c*_i,f_ is the steady-state *c*_i_ approximated as 0.65*c*_a_, and *R*_d_ was assumed to be 1.6 µmol m^−2^ s^−1^ (the fitted value from our steady-state *A*/*c*_i_ response). *A*_i_ is the gross assimilation extrapolated to *t* = 0, which provides an estimate of initial Rubisco activation [3,23]. Finally, *τ* is the time constant for recovery of photosynthesis. The model was fit using both nonlinear least squares (using data collected from 60 s until 600 s after the change in PPFD), and the linear regression technique described previously [23], where a plot of 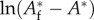 against time has slope 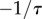 and intercept 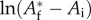. The same model was fit to A* and *V_c_*_,max_, allowing a novel comparison between estimates of *τ* for Rubisco activation based on A* and *V_c_*_,max_.

To obtain integrated CO_2_ assimilation (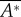) during increases in PPFD, if it is assumed that RuBP concentration is saturating, the model can be re-written as [24]

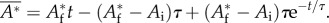


Setting *τ* = 0 estimates potential assimilation rate with a square response to PPFD (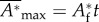), and an estimate of foregone assimilation is 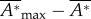. To determine the impacts of Rubisco kinetics on CO_2_ assimilation, the response of 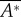 to PPFD was modelled at approximately 60 s time intervals during a diurnal period. A PPFD regime was used that predicted light available to the second layer of a crop canopy [25]. This is justified by the observation that the ears represent the first layer and cause intermittent shading of the flag leaves as the angle of the sun progresses through the day. In the data used, PPFD at a point on the leaf had been predicted using reverse ray tracing, with shade-generating structures in the canopy distributed at random within each layer. A clear sky day in June at latitude 44°N had been assumed for calculating sun angles over the course of the day [25]. To model gross photosynthesis throughout the diurnal period, initial photosynthesis for each approximately 60 s interval (*A*_i_) was taken to be A* predicted for the preceding interval, except at first light where *A*_i_ was assumed to be zero. The potential maximum gross rate of photosynthesis during each timestep (A*_f_) was predicted as 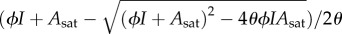, using parameters from the PPFD response curves fit to steady-state data and setting *t* = duration of the timestep (s). When PPFD was increasing we set *τ* to 180 s, the mean value determined by substituting the time-series of *V_c_*_,max_ from our dynamic *A*/*c*_i_ analysis into the induction model: 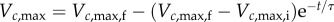. When PPFD was decreasing, we estimated 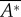 as 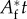, and predicted *A*_i_ as above, but using *τ* = 300 s for the rate of decrease towards the lower A*_f_ predicted from PPFD. The value of *τ* = 300 s for the decrease was predicted on the basis that 30 min ‘shade’ treatment resulted in a decrease in *V_c_*_,max_ from 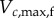 to 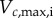.

## 3. Results

### (a) Factors limiting photosynthesis in wheat, cv. Highbury: steady state

**Figure 1.**
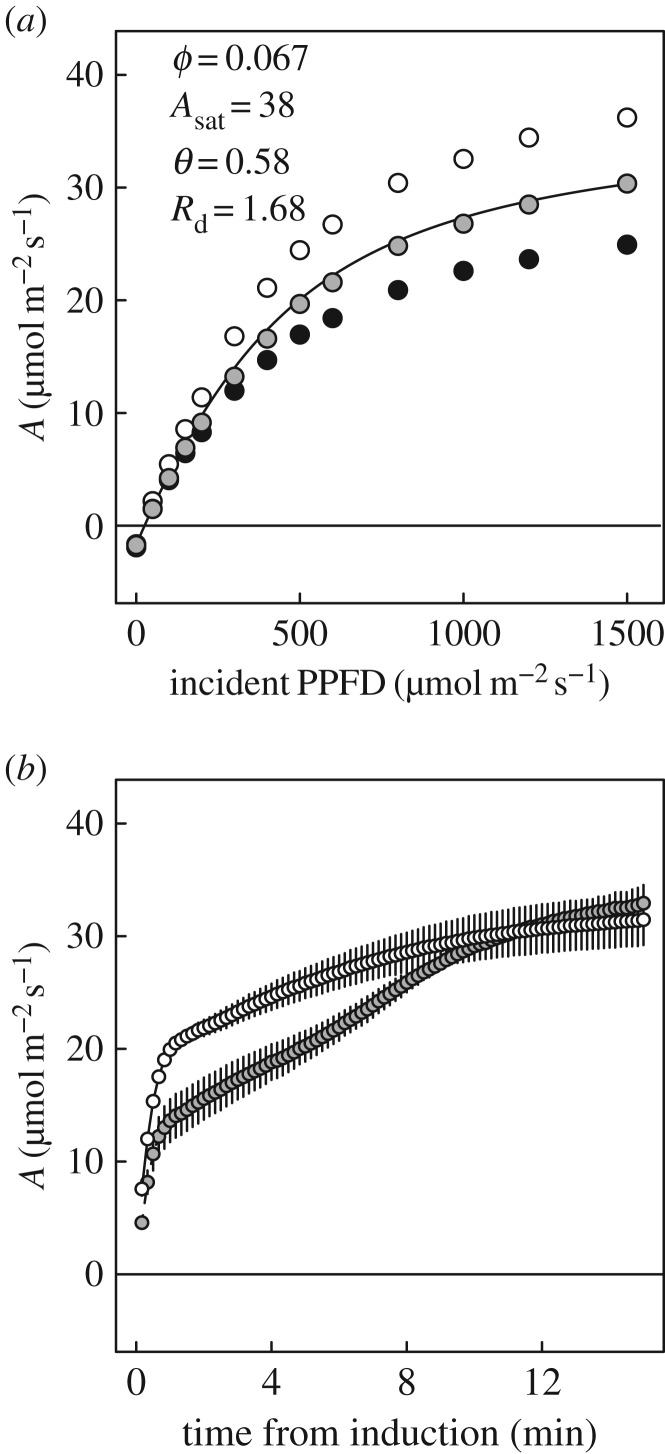
Responses of photosynthetic CO_2_ uptake (*A*) to PPFD in flag leaves of bread-wheat at heading-anthesis. (*a*) Static light-response: solid line indicates light response based on means of fitted parameters shown, symbol shading differentiates three plants used in the experiment. (*b*) Dynamics of photosynthetic induction following a transition from 50 to 1200 µmol m^−2^ s^−1^ PPFD (shade to sun): values are means ± s.e. based on three leaves from separate plants, shaded symbols and dashed lines indicate leaves maintained at 400 µmol mol^−1^ [CO_2_] during the preceding 30 min shade period, open symbols and solid lines indicate leaves maintained at 100 µmol mol^−1^ [CO_2_] during the preceding 30 min shade period.

**Figure 2.**
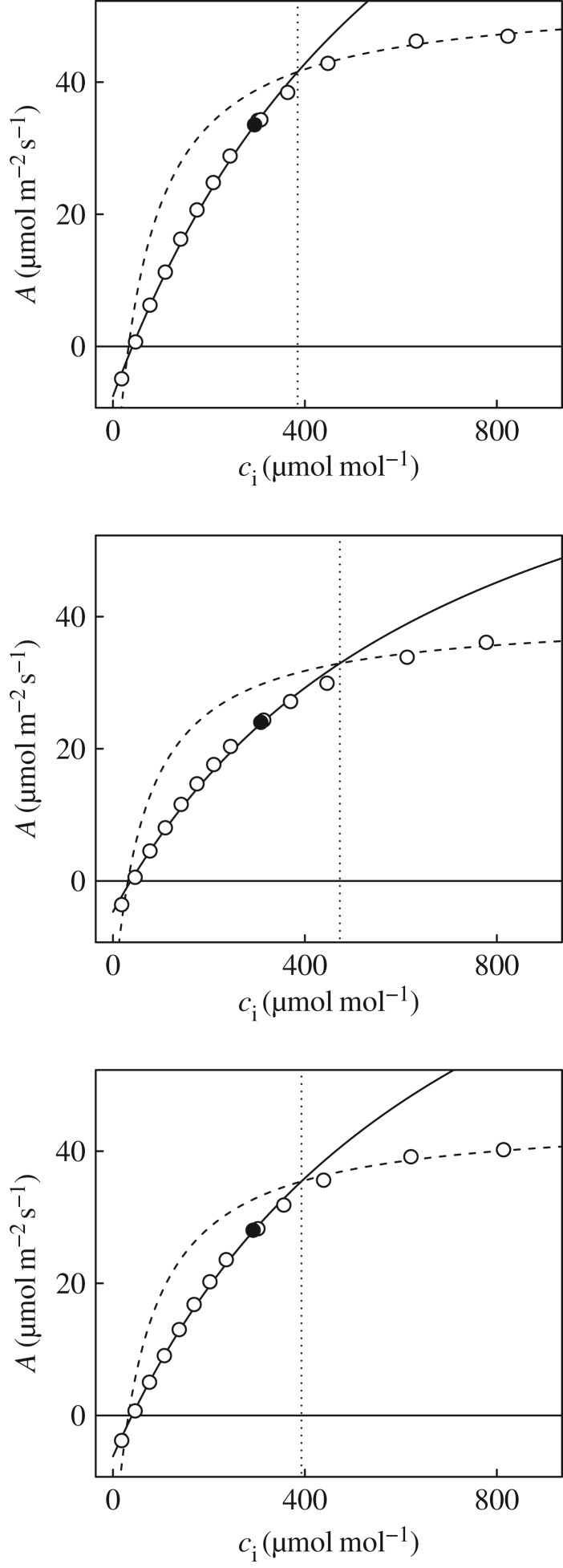
Response of net leaf CO_2_ uptake (*A*) to intercellular CO_2_ concentrations (*c*_i_) in flag leaves of wheat at heading-anthesis. Fitted curves are shown for Rubisco-limited photosynthesis (solid lines) and RuBP-limited photosynthesis (dashed lines). Vertical dotted lines indicate the *c*_i_ at which limitation of photosynthesis transitions from Rubisco to RuBP regeneration (*c*_i,trans_), and filled points the steady-state operating values. Parameter values were (mean ± s.e., *N* = 3): *V_c_*_,max_ = 113 ± 12.7 µmol m^−2^ s^−1^; *R*_d_ = 1.6 ± 0.31 µmol m^−2^ s^−1^; *J* = 214 ± 18.3 µmol m^−2^ s^−1^; *Γ* = 39.0 ± 1.00 µmol mol^−1^; *c*_i,trans_ = 407 ± 27.4 µmol mol^−1^. Conditions during measurements for each leaf were as follows (mean, CV < 0.01): vapour pressure deficit, 0.99 kPa; photosynthetic photon flux density, 1200 µmol m^−2^ s^−1^; leaf temperature, 25°C.

Responses to light and CO_2_ measured from steady-state photosynthesis indicated high maximum net leaf CO_2_ assimilation rates (*A*_sat_ > 30 µmol m^−2^ s^−1^; figures 1 and 2), with saturation approached at a PPFD of about 1200 µmol m^−2^ s^−1^ (figure 1*a*). This was the subsequent level chosen as a proxy for ‘sun’ conditions in examining induction. On transfer from ‘shade’ (50 µmol m^−2^ s^−1^) to ‘sun’ at *c*_a_ of 400 µmol mol^−1^ there was an initial rapid increase in *A* (figure 1*b*), followed by a slower increase lasting *ca* 15 min. When leaves were maintained at a *c*_a_ of 100 µmol mol^−1^ in the shade to prevent stomatal closure, then exposed to ‘sun’ at *c*_a_ 400 µmol mol^−1^, the initial transient increase in *A* saturated at a higher value, indicating a decrease in stomatal limitation; however, after 10 min *A* was similar in the two experiments (figure 1*b*).

Static *A/c*_i_ responses showed that at steady state, limitation of *A*_sat_ was consistent with *A*_C_ (figure 2); *c*_i,trans_, the transition from limitation by *A*_C_, with *V_c_*_,max_ (113 ± 13 µmol m^−2^ s^−1^; mean ± s.e., *N* = 3), to limitation by *A*_J_, with *J* (214 ± 18 µmol m^−2^ s^−1^), occurred at 407 ± 27 µmol mol^−1^. This transition was therefore well above the operating *c*_i_, i.e. that obtained at the current atmospheric level of 400 µmol mol^−1^ and above the *c*_i_ that would be obtained under the slightly elevated ambient *c*_a_ in the greenhouse of 449 µmol mol^−1^ (figure 2). Stomatal limitation to *A*_sat_ at 400 µmol mol^−1^ *c*_a_ (*l*) was 0.196 ± 0.010 (mean ± s.e.), i.e. if there was no diffusive barrier at the epidermis *A*_sat_ would be about 20% higher.

### (b) Factors limiting photosynthesis in wheat, cv. Highbury: during induction

**Figure 3.**
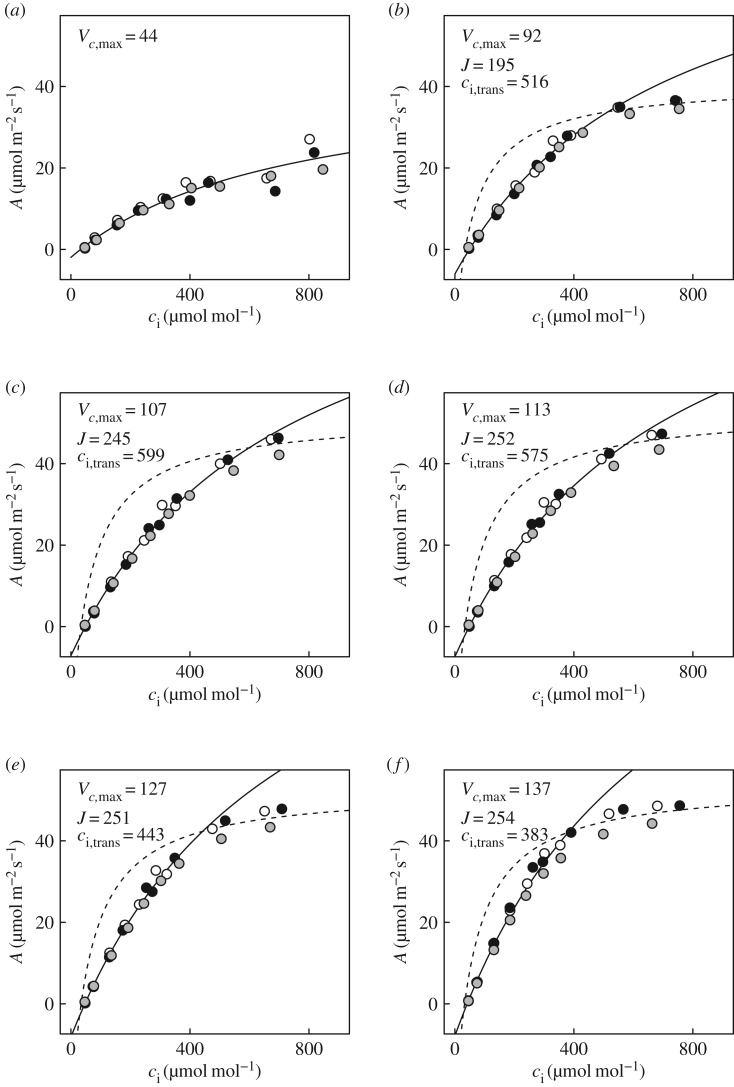
Photosynthetic induction after transition from 50 to 1200 µmol m^−2^ s^−1^ PPFD, represented by dynamic *A*/*c*_i_ analysis at: (*a*) 20 s; (*b*) 1 min; (*c*) 2.5 min; (*d*) 3 min; (*e*) 4.5 min; (*f*) 10 min.

*A*/*c*_i_ responses constructed for each 10 s interval of induction following transition from 50 to 1200 µmol m^−2^ s^−1^ PPFD (electronic supplementary material, figure S1) showed several phases of photosynthetic limitation. Admissible, best fitting models during the first 40 s after the transition to sun, consisted in most cases solely of limitation by *A*_C_, with *V_c_*_,max_ at less than 40% of its steady-state value (compare figures 2 and 3*a*). However, sums of squares (SS) for residuals of models fit as a single limitation phase were relatively high (6.97–37.91); stronger fits were obtained when both *A*_C_ and *A*_J_ could be identified (figure 3*b–f*; SS, 1.04–12.29).

Initially, both *V_c_*_,max_ and *J* increased, but *J* increased more rapidly than *V_c_*_,max_, so *c*_i,trans_ rose to a maximum approaching 600 µmol mol^−1^ at around 2.5 min (figure 3*b,c*). At 3 min, *J* saturated close to 250 µmol m^−2^ s^−1^ and *c*_i,trans_ began to decrease as *V_c_*_,max_ slowly rose (figure 3*d*). For the remainder of the first 10 min following the transition, decreases in *c*_i,trans_ continued, in concert with increasing *V_c_*_,max_. The increase in *V_c_*_,max_ was most rapid in the first 4.5 min (figure 3*e*), and adjustment continued through to 10 min (figure 3*f*). After this time, *A*/*c*_i_ responses were comparable with those measured at steady state (figures 2 and 3*f*).

**Figure 4.**
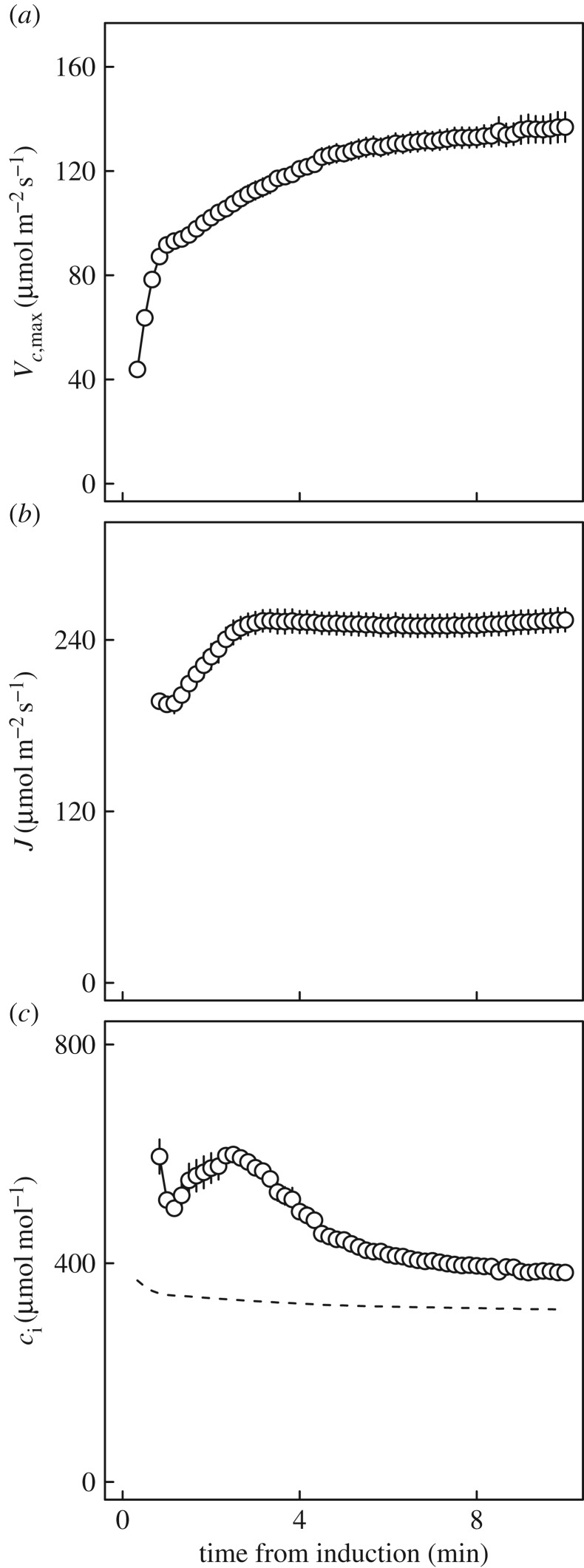
Dynamics of photosynthetic limitations affecting wheat leaves over 10 min following a step change in PPFD from 50 to 1200 µmol m^−2^ s^−1^ (shade to sun). (*a*) Maximum rate of Rubisco carboxylation (*V_c_*_,max_). (*b*) Rate of electron transport (*J*). (*c*) The *c_i_* at which the primary limitation imposed on photosynthesis switches between *V_c_*_,max_ and *J* (*c*_i,trans_). Values are means ± s.e. based on three leaves from separate plants (indicated by symbol shading). The dashed line in (*c*) places an upper limit on operating *c*_i_, assuming a chamber [CO_2_] (*c*_a_) of 400 µmol mol^−1^, and steady-state stomatal conductance of 0.7 mol m^−2^ s^−1^.

Time series for *V_c_*_,max_, *J* and *c*_i,trans_ (figure 4) developed from data including those shown in figure 3, provided a *τ* for *V_c_*_,max_ of *ca* 3 min (mean ± s.e.m., 181 ± 12.8 s), more than three times that for *J* (50.1 ± 1.91 s); slow adjustment in *V_c_*_,max_ clearly had a strong effect on *c*_i,trans_ between 2.5 and 10 min into the induction (figure 4). Calculation of a maximum probable operating *c*_i_ (figure 4; based on *A*/*c*_i_ responses and steady state *g*_s,w_) further demonstrated that *c*_i,trans_ exceeded this value throughout the period of induction, confirming that in our analysis apparent *V_c_*_,max_ was the dominant biochemical variable limiting photosynthesis through the induction (figure 4*c*). Comparisons of *τ* for *V_c_*_,max_ with estimates of *τ* for Rubisco activation effects on photosynthesis based on A* suggested a range of values for *τ* between 3 and 4 min (electronic supplementary material, figure S2).

**Figure 5.**
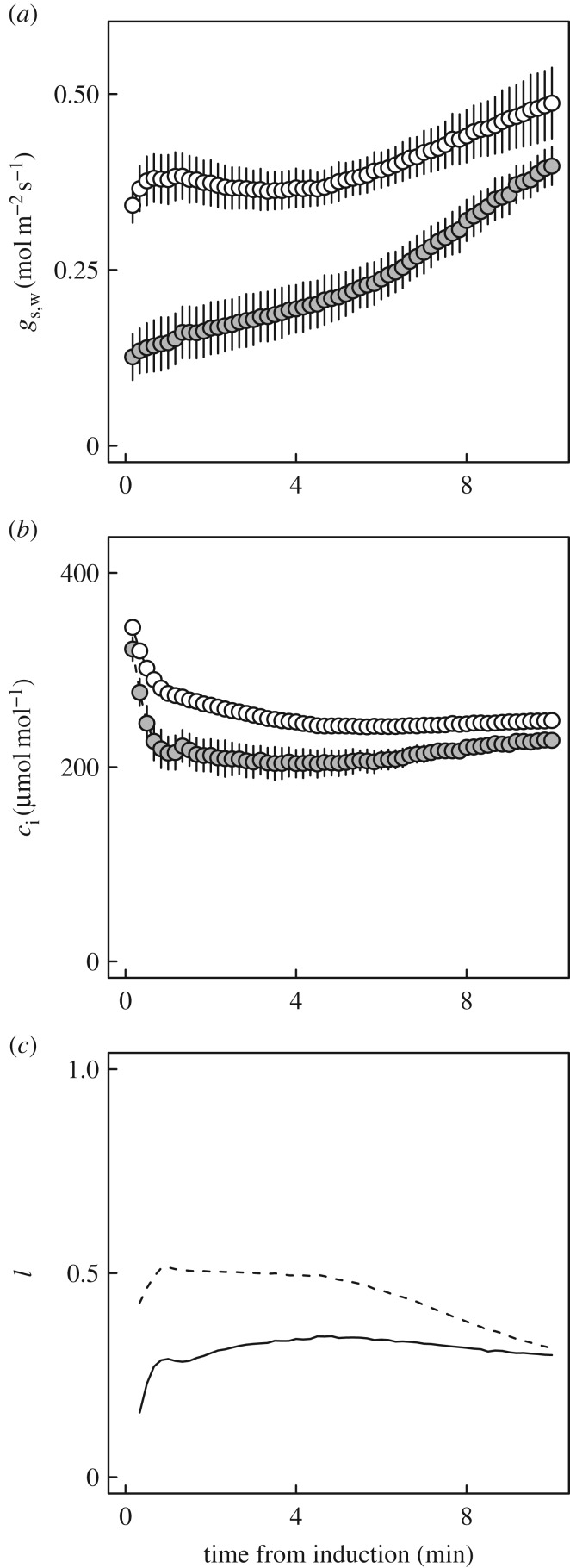
Stomatal effects following a step change in PPFD from 50 to 1200 µmol m^−2^ s^−1^, as affected by [CO_2_] pre-treatment. (*a*) Stomatal conductance to water vapour (*g*_s,w_). (*b*) Intercellular CO_2_ concentrations (*c_i_*). Values are means ± s.e. based on three leaves from separate plants. (*c*) Limitation imposed by stomata (*l*), relative to infinite conductance at [CO_2_] = 400 µmol mol^−1^. Shaded symbols and dashed lines are for 400 µmol mol^−1^ [CO_2_] during shade; open symbols and solid lines are for 100 µmol mol^−1^ [CO_2_] during shade.

Shading, such as that simulated by our 50 µmol m^−2^ s^−1^ PPFD pre-treatment, affects stomatal opening. To characterize *A*_J_ using dynamic *A*/*c*_i_ analysis, it was necessary to increase stomatal conductance following shade by decreasing *c*_a_ during the low-light pre-treatment. Compared with plants pre-treated at 400 µmol mol^−1^ *c*_a_, during the first 4 min after illumination both *A* and *g*_s,w_ of plants pre-treated at *c*_a_ = 100 µmol mol^−1^ were higher by 30–65% (figure 1*b*) and 88–171% (figure 5*a*), respectively, resulting in an increase in cumulative net CO_2_ assimilation of 22%. Pre-treatment with a *c*_a_ of 100 µmol mol^−1^ also resulted in progressive decreases in *c*_i_ through the induction, suggesting increasing photosynthetic efficiency was a key control on *c*_i_ (figure 5*c*). Pre-treatment at *c*_a_ = 400 µmol mol^−1^ resulted in rapid declines of *c*_i_ to a minimum that was maintained for around 5 min before *c*_i_ started to increase (figure 5*b*). In both cases, after 10 min *c*_i_ remained below values expected at steady state (figures 2 and 4*c*); slow relaxation of stomatal limitation affected *c*_i_ over considerably longer periods than relaxation of limitation by *V_c_*_,max_. Immediately after PPFD increased, *l* in leaves pre-treated at a *c*_a_ = 400 µmol mol^−1^ CO_2_ was twice as high as in leaves treated at 100 µmol mol^−1^, reaching a maximum of 0.5. After 10 min *l* was similar between the two treatments (figure 5*d*), but remained 50% higher than for steady state *A*/*c*_i_ responses in both cases (400 µmol mol^−1^, 0.32; 100 µmol mol^−1^, 0.3).

### (c) Impact of induction characteristics on diurnal photosynthesis

**Figure 6.**
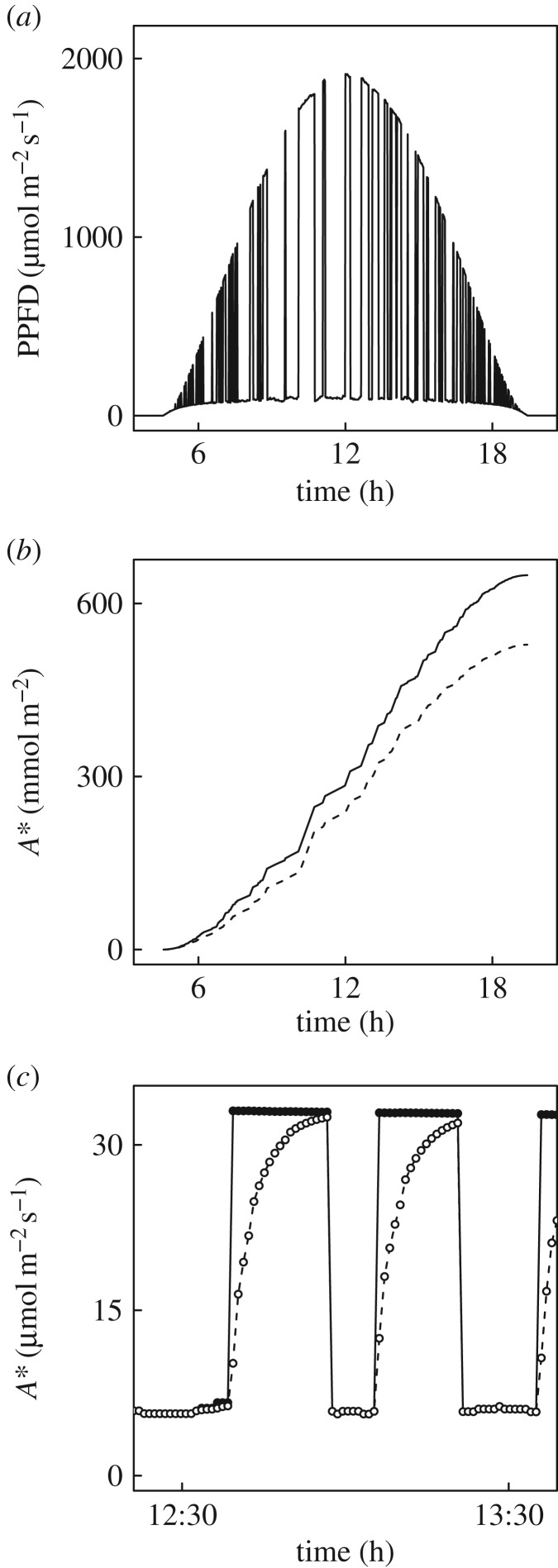
(*a*) The simulated course of photon flux (PPFD) on a clear sky June day at latitude 44°N for a point on the flag leaf, assuming one layer of randomly distributed elements above the leaf [25]. (*b*) The cumulative gross assimilation of CO_2_ assuming that A* instantaneously adjusts to the steady-state value fit to the light response curve (figure 1*a*), i.e. no lag on a shade to sun transition (solid line); versus accounting for the lag imposed by Rubisco re-activation (dashed line). (*c*) As for (*b*) but showing instantaneous A* for the period around solar noon for the no-lag scenario (filled symbols) and the scenario modelled on measured Rubisco activation (open symbols).

Simulation of PPFD fluctuations that would occur at a single point on a flag leaf due to intermittent shading from ears and other flag leaves on a clear sky summer day, shows multiple transitions from shade to sun and back to shade (figure 6*a*). Based on the response of *A* to PPFD determined for these leaves (figure 1*a*), the cumulative assimilation of CO_2_ over the course of a clear sky day, accounting for the fluctuations in PPFD, is shown in the upper line of figure 6*b*. The total uptake of CO_2_ over the daylight hours is 640 mmol m^−2^. When account is taken of de-activation of Rubisco, depending on duration of the ‘shade’ period and then subsequent re-activation, cumulative CO_2_ assimilation would follow the lower line. This reaches a total of 506 mmol m^−2^ or a 21% reduction due to the slow recovery of photosynthetic efficiency, due to the re-activation of Rubisco, following shade to sun transitions. The dynamics of this loss may be seen more clearly from a narrower time window around solar noon. Here, for instantaneous assimilation rates the area above the dotted line and below the solid line represents lost assimilation (figure 6*c*).

## 4. Discussion

On shade to sun transitions, this study has shown that several minutes are required for the wheat flag leaf to re-attain maximum photosynthetic efficiency (figure 1*b*). At the level of leaf biochemical limitations, the apparent maximum activity of Rubisco (*V_c_*_,max_) limits this rate of induction, implying activation of this enzyme as the key factor, rather than regeneration of the RuBP CO_2_ acceptor molecule (*J*). This was clearly indicated by the fact that *c*_i,trans_ was well above the actual *c*_i_ when *c*_a_ was at the current atmospheric level of 400 µmol mol^−1^ and at the actual greenhouse growth *c*_a_ of 449 µmol mol^−1^ (figures 3 and 4*c*). In contrast to previous studies [4], stomatal limitation plays a role in the speed of induction, declining from *ca* 0.5 in the first 3 min to about 0.3 at steady state, indicating that about 20% of the lag is due to stomatal movement (figure 5*c*). This is also indicated by the fact that when the leaf is at the ambient *c*_a_ of 400 ppm throughout, *c*_i_ declines to about 200 µmol mol^−1^ before recovering to *ca* 230 µmol mol^−1^ at steady state (figure 5*b*). Combining the ray tracing model of Zhu *et al*. [25] and the modelled kinetics of Rubisco de-activation and activation on sun-to-shade-to-sun transitions following Woodrow *et al*. [3,23,24], losses due to the slow induction were calculated. Parametrized on the data reported here for wheat flag leaves, the lag in activation of Rubisco following shade to sun transitions resulted in a 21% loss of potential flag leaf assimilation (figure 6).

The findings (figures 3 and 4*c*) indicate *V_c_*_,max_, or the apparent maximum activity of Rubisco, as the major factor limiting the rate of induction, implying the speed of re-activation of Rubisco. This is consistent with previous studies of tobacco, rice and soya bean [3–5]. However, the apparent *V_c_*_,max_ calculated from the *A*/*c*_i_ response is also affected by mesophyll conductance (*g*_m_). [CO_2_] at Rubisco (*c*_c_) will be less than *c*_i_ due to mesophyll conductance. If *g*_m_ increased during the course of induction, it would cause part of the apparent increase in *V_c_*_,max_. As a physical conductance, *g*_m_ would not vary. However, modelling suggests that in reality it will have some dependence on the positioning of organelles, and in particular the relative localization of chloroplasts and mitochondria, which may change in response to light levels within the leaf [26–28]. It is known that chloroplasts may alter their position with PPFD. Through its impact on *g*_m_, this movement could explain some, but certainly not all, of the change in apparent *V_c_*_,max_ [28]. Transporters and channels in membranes may change dynamically to affect *g*_m_. Therefore, the lag attributed to Rubisco activation could in reality be a combination this activation with an increase in *g*_m_.

Previous research has shown a strong correlation between the speed of induction and the activation of Rubisco, in particular, the enzyme Rubisco activase [3,5]. Also, as noted above, in contrast to a previous dynamic analysis of *A*/*c*_i_ responses in induction in soya bean [4], stomata limit the speed of induction, accounting for about 20% of the change (figure 5). However, stomatal opening appears to depend strongly on photosynthesis in the mesophyll [29,30]. Thus, there may be some dependency of the speed of stomatal opening on the speed of Rubisco activation in the mesophyll. Assuming *c*_c_ in the shade is sufficient to support rapid carbamylation of Rubisco, increasing the speed of activation might increase the speed of stomatal opening.

The dynamic *A*/*c*_i_ method used to identify photosynthetic limitations in this study has been developed recently [4]. In this study, we found that it was necessary to decrease *c*_a_ in the ‘shade’ in order to limit stomatal closure that otherwise prevented characterization of *A*_J_ in wheat. We anticipate that this technical solution will not have had a substantial effect on Rubisco activation independent of the ‘shade’ because at low light photosynthesis will be entirely limited by RuBP regeneration not Rubisco, and because *c*_i_ remained high. Decreases in activation linked with de-carbamylation as a result of low CO_2_ availability [31] are unlikely in this scenario. Perhaps more importantly, dynamic *A*/*c*_i_ analyses are intended to capture non-steady state dynamics, and do so by characterizing induction at a range of *c*_a_. The rate of Rubisco activation during induction is thought to respond to CO_2_ availability [32], consistent with greater availability of CO_2_ driving Rubisco carbamylation and minimizing alternative reactions (reviewed in [33]). The timed snapshots obtained using dynamic *A*/*c*_i_ analysis, in strict terms, violate the usual assumption made when using the Farquhar *et al*. model [34] that Rubisco activity is at steady state. Calculating *V_c_*_,max_ in a dynamic analysis averages across measurements that may reflect different activation states. It is also possible that the eventual steady state of activation during each induction will depend on *c*_a_, but evidence suggests decreases in activation under light saturated conditions are usually observed only when *c*_i_ is significantly below 100 µmol mol^−1^, and then only in certain species [31]. Nonetheless, specific parameter values for dynamic *A*/*c*_i_ response curves should be interpreted with some caution. The usefulness of the dynamic *A*/*c*_i_ analysis is primarily as a mean of assessing the sequence and approximate timing of transitions between different photosynthetic limitations during induction. We anticipate that experimentation and modelling to understand how *c*_a_ affects Rubisco activation state during induction will improve our understanding of the induction process, and the potential feedbacks due to mesophyll and stomatal conductance responses.

Importantly, this research shows that the speed of non-steady-state adjustment of photosynthesis to light fluctuations in the field, regardless of underlying cause, will strongly affect flag leaf photosynthesis. In turn, this will decrease the supply of assimilate for the developing grain. Although, only the flag leaf was examined here, the same lags in induction will likely apply to all leaves of the plant. Thus, the growth and production that supports the development of the plant to flowering and seed fill will be affected. Increasing the rate of induction following shade to sun transitions under typical field conditions during grain filling would decrease the impact of a significant limitation, and therefore represents an excellent target through which increases in productivity would be obtained. The gains in productivity could be of similar magnitude to those observed by bioengineering a faster rate of adjustment to sun to shade transitions [1]. Acceleration might be achieved by over-expressing the amount of Rca [5], by targeted amino acid substitutions of Rca [35], altered ratios of alpha and beta forms [35], or by exploring the natural variation in speed of adjustment apparent in soya bean [4]. The results presented here suggest that these changes have the potential to open an important new route, through photosynthesis, to a much needed yield jump for wheat.

## Supplementary Material

Original data from each plant sampled

## Data accessibility

The datasets supporting this article are available from Lancaster University: doi:10.17635/lancaster/researchdata/144.
