# Supplementary material for: Slow induction of photosynthesis on shade to sun transitions in wheat may cost at least 21% of productivity
Source: Philos Trans R Soc Lond B Biol Sci. 2017 Aug 14;372(1730):20160543. doi: 10.1098/rstb.2016.0543 (PMC5566890; doi:10.1098/rstb.2016.0543)
Supplement: Original data from each plant sampled [file rstb20160543supp1.pdf]

Supplementary Information: Philosophical Transactions of the Royal Society B

Slow induction of photosynthesis on shade to sun transitions in wheat may cost at least 21% of productivity.

Samuel H. Taylor[1], Stephen P. Long[1,2]

[1]Lancaster Environment Centre, Lancaster University, United Kingdom LA1 4YQ, UK

[2*]Departments of Crop Sciences and Plant Biology, University of Illinois, Urbana, IL 61801, USA

*For correspondence slong@illinois.edu

Contents:

Figure 1. A-c_i responses fitted to gas exchange at different c_a (50, 100, 200, 300, 400, 500, 600, 800, 1000 µmol mol^{-1}) measured at 10 s intervals during 10 min after transitions from 50 µmol m^{-2} s^{-1} PPFD and 100 µmol mol^{-1} CO_2, to 1200 µmol m^{-2} s^{-1} PPFD and the target c_a. Symbols show measured net CO_2 assimilation (A) and intercellular [CO_2] (c_i). Solid red curves represent Rubisco limited photosynthesis; solid green curves represent RuBP-regeneration limited photosynthesis. Vertical dotted lines show CO_2 compensation points (Γ); vertical dashed lines indicate the c_i at which limitation of A would transition from Rubisco to RuBP regeneration ($c_{i,trans}$). Curves shown represent three dynamic A/c_i responses measured on separate plants, indicated as ST1, ST2, and ST3.

Figure 2. Dynamic responses of A^* during 10 min after transitions from 50 to 1200 µmol m^{-2} s^{-1} PPFD. Mean values (N = 3) were predicted based on: dotted line, non-linear least squares fits of $A^* = A^*_f - (A^*_f - A_i)e^{-t/\tau}$; dashed line, linear fits to $\ln(A^*_f - A^*)$, of which the slope is $-1/\tau$; solid line, non-linear least squares fits of $V_{cmax} = V_{cmax,f} - (V_{cmax,f} - V_{cmax,i})e^{-t/\tau}$, followed by prediction of $A^* = V_{cmax}(c_i - \Gamma^*/c_i + K_{CO})$, where Γ^* and K_{CO} were taken to be the values at 25 °C based on Bernacchi *et al*., (2003) *Plant Cell and Environment* **26**, 1419-1430.

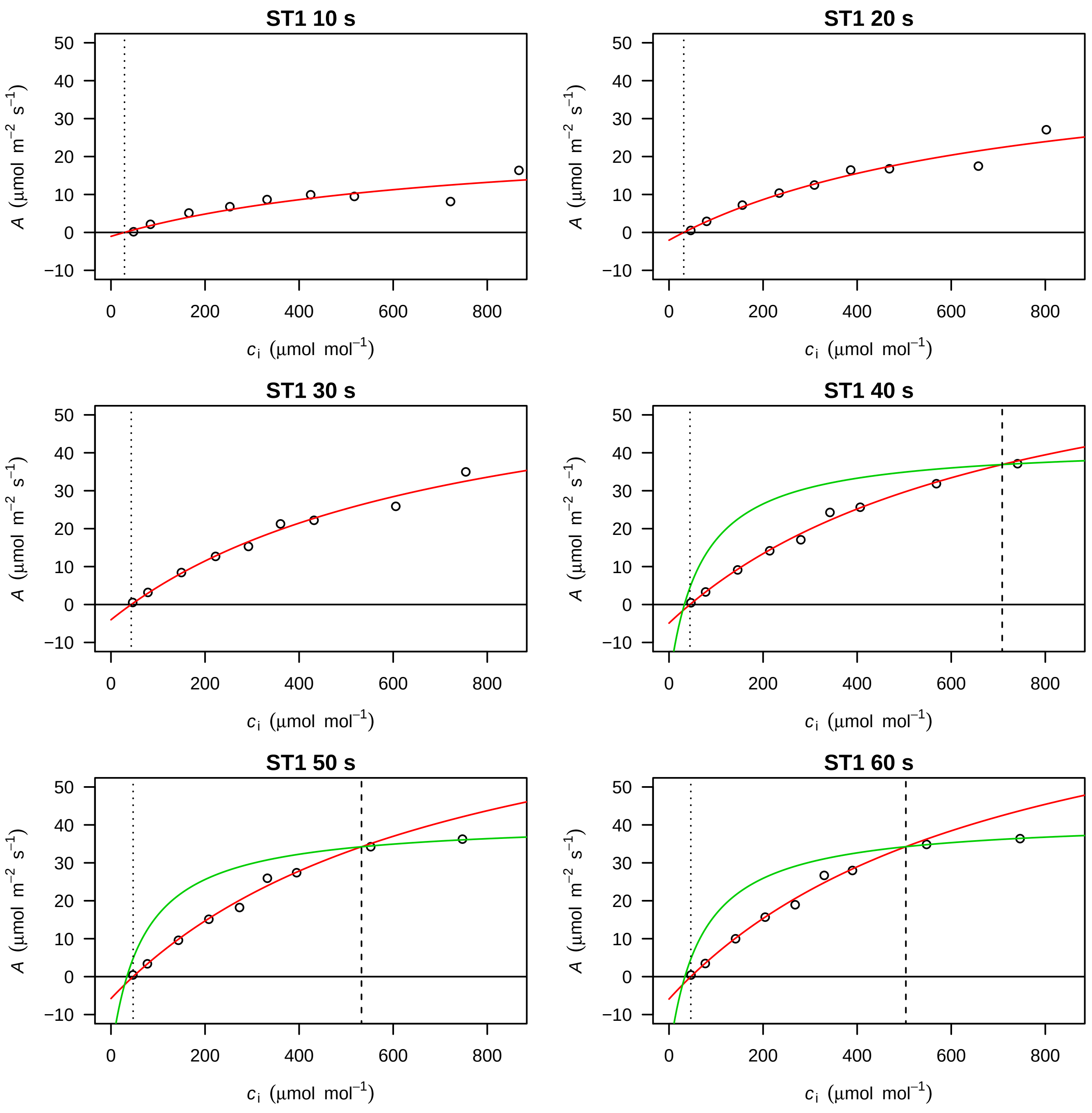
ST1 10 s
ST1 20 s
ST1 30 s
ST1 40 s
ST1 50 s
ST1 60 s
A (µmol m−2 s−1)
ci (µmol mol−1)
50
40
30
20
10
0
−10
0
200
400
600
800

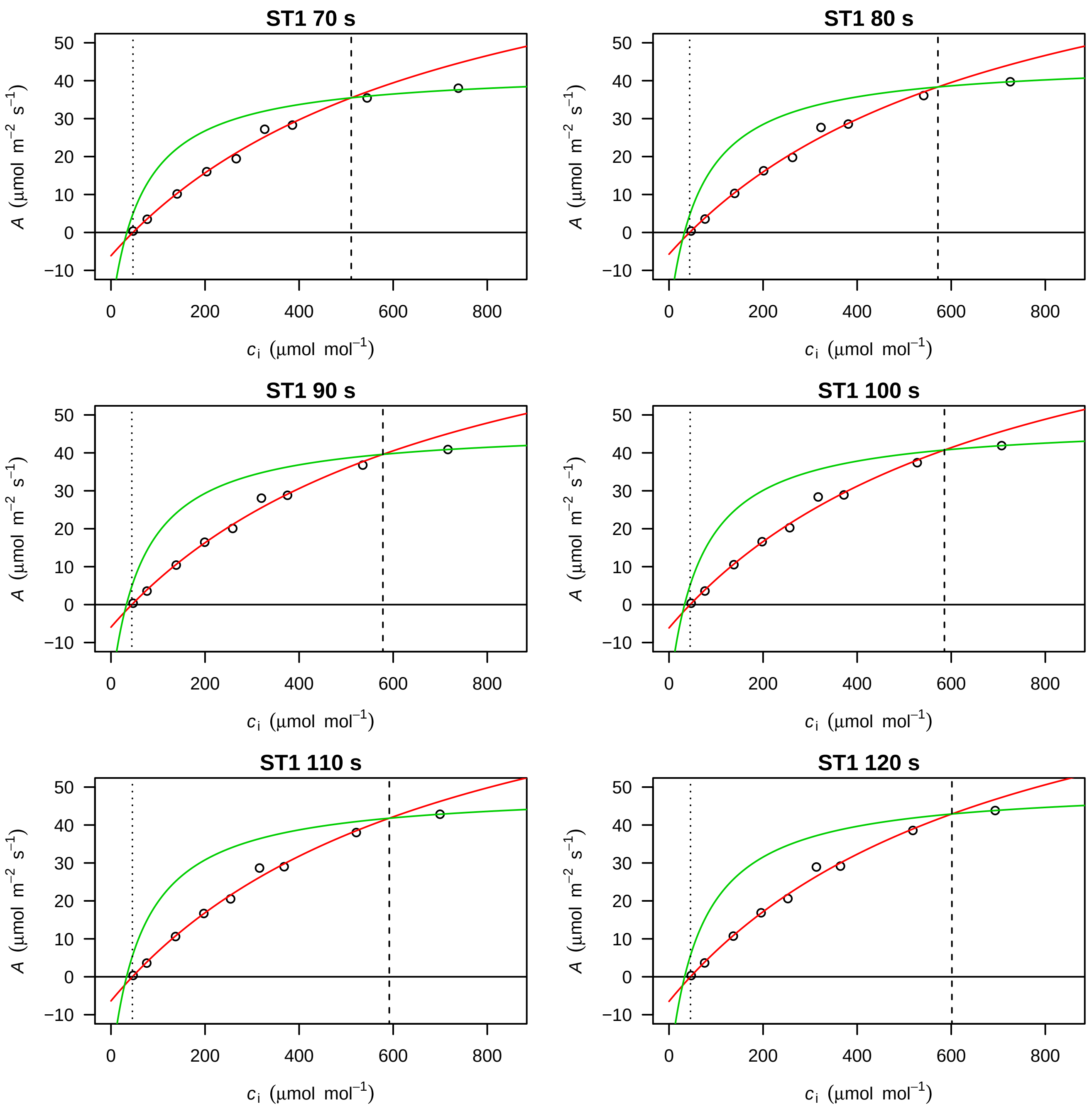

ST1 70 s
ST1 80 s
ST1 90 s
ST1 100 s
ST1 110 s
ST1 120 s
A (µmol m−2 s−1)
ci (µmol mol−1)

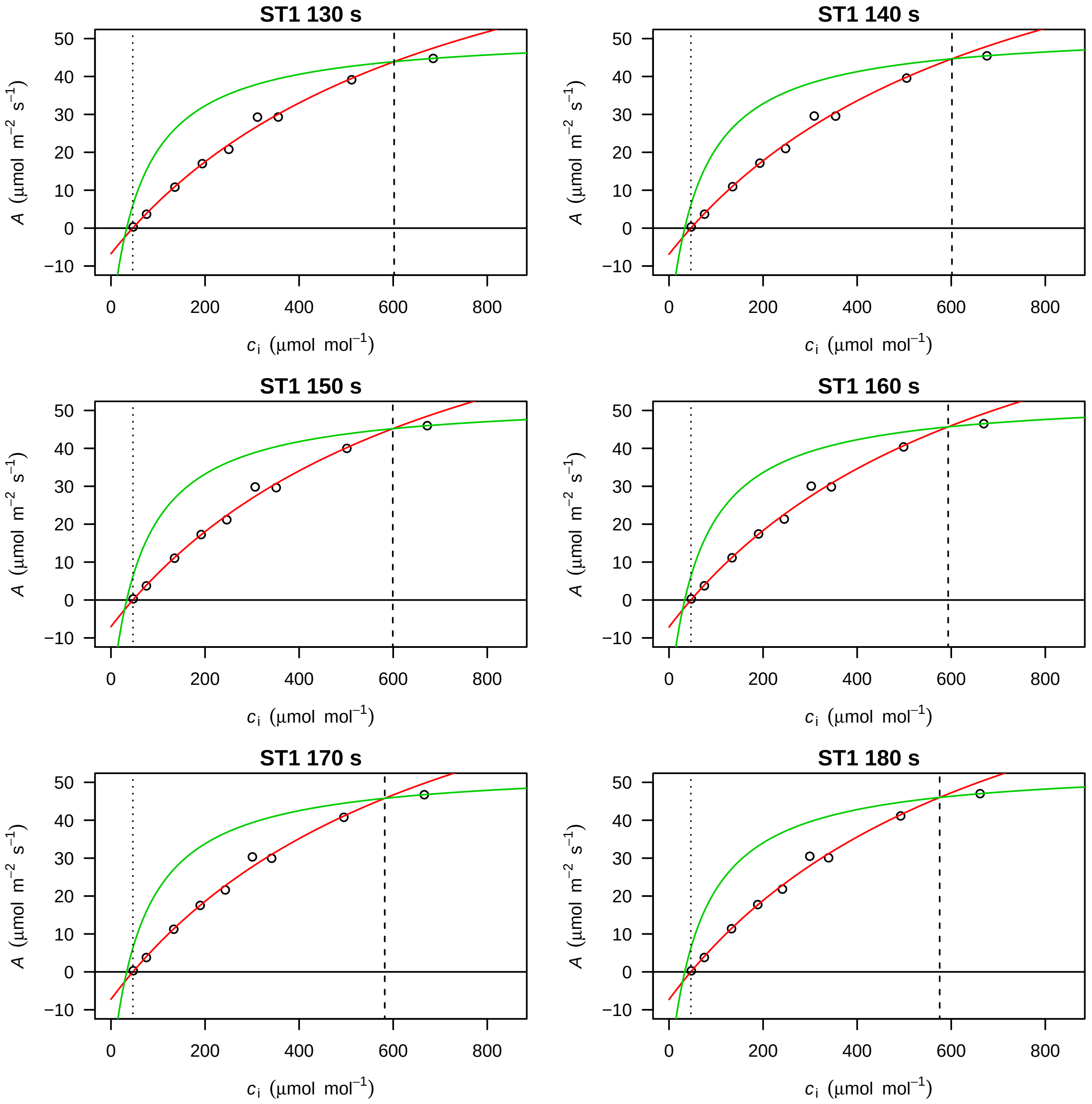

ST1 130 s
ST1 140 s
ST1 150 s
ST1 160 s
ST1 170 s
ST1 180 s
A (µmol m^{-2} s^{-1})
c_i (µmol mol^{-1})

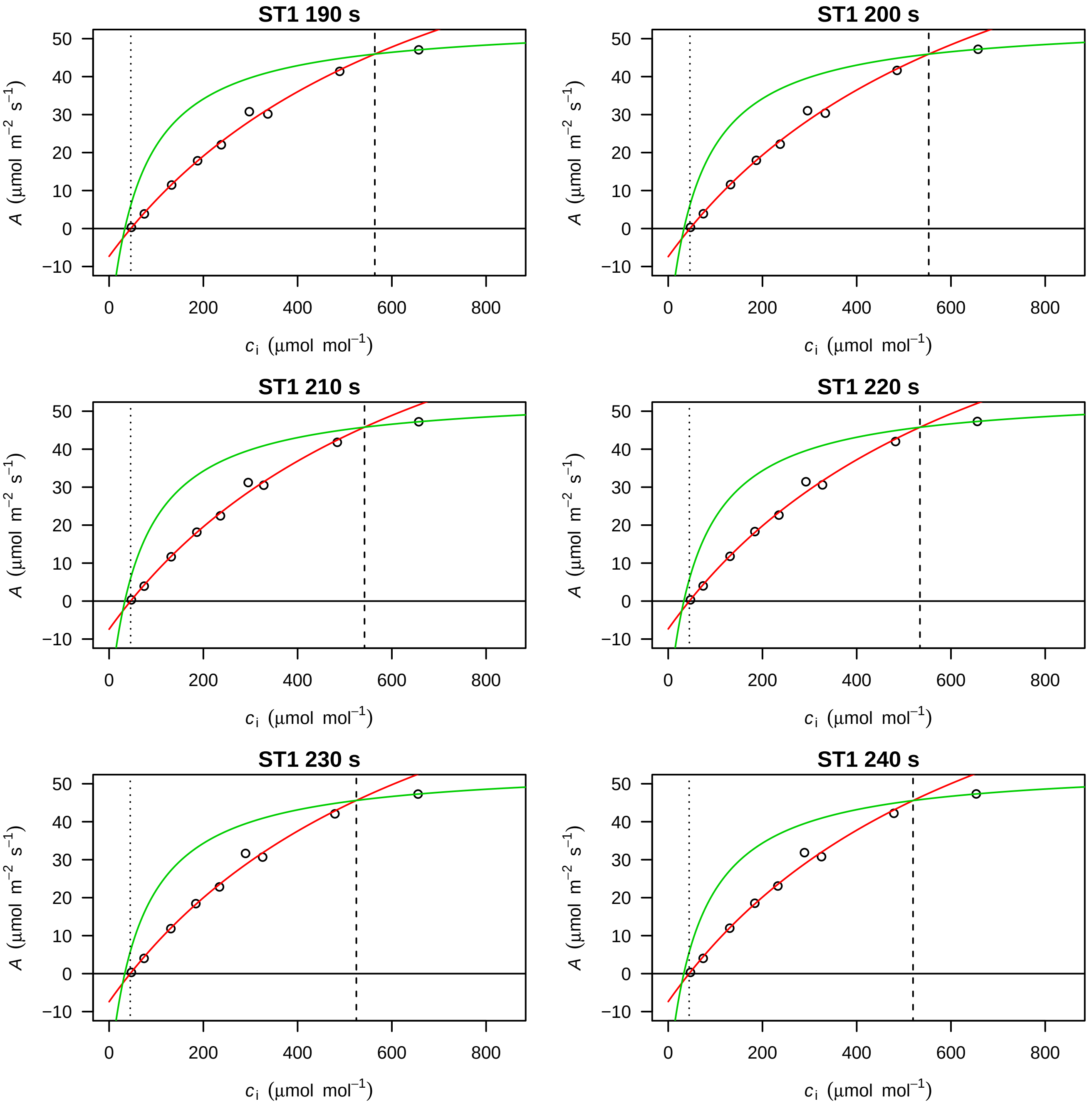
ST1 190 s
ST1 200 s
ST1 210 s
ST1 220 s
ST1 230 s
ST1 240 s
A (μmol m−2 s−1)
ci (μmol mol−1)
50
40
30
20
10
0
−10
0
200
400
600
800

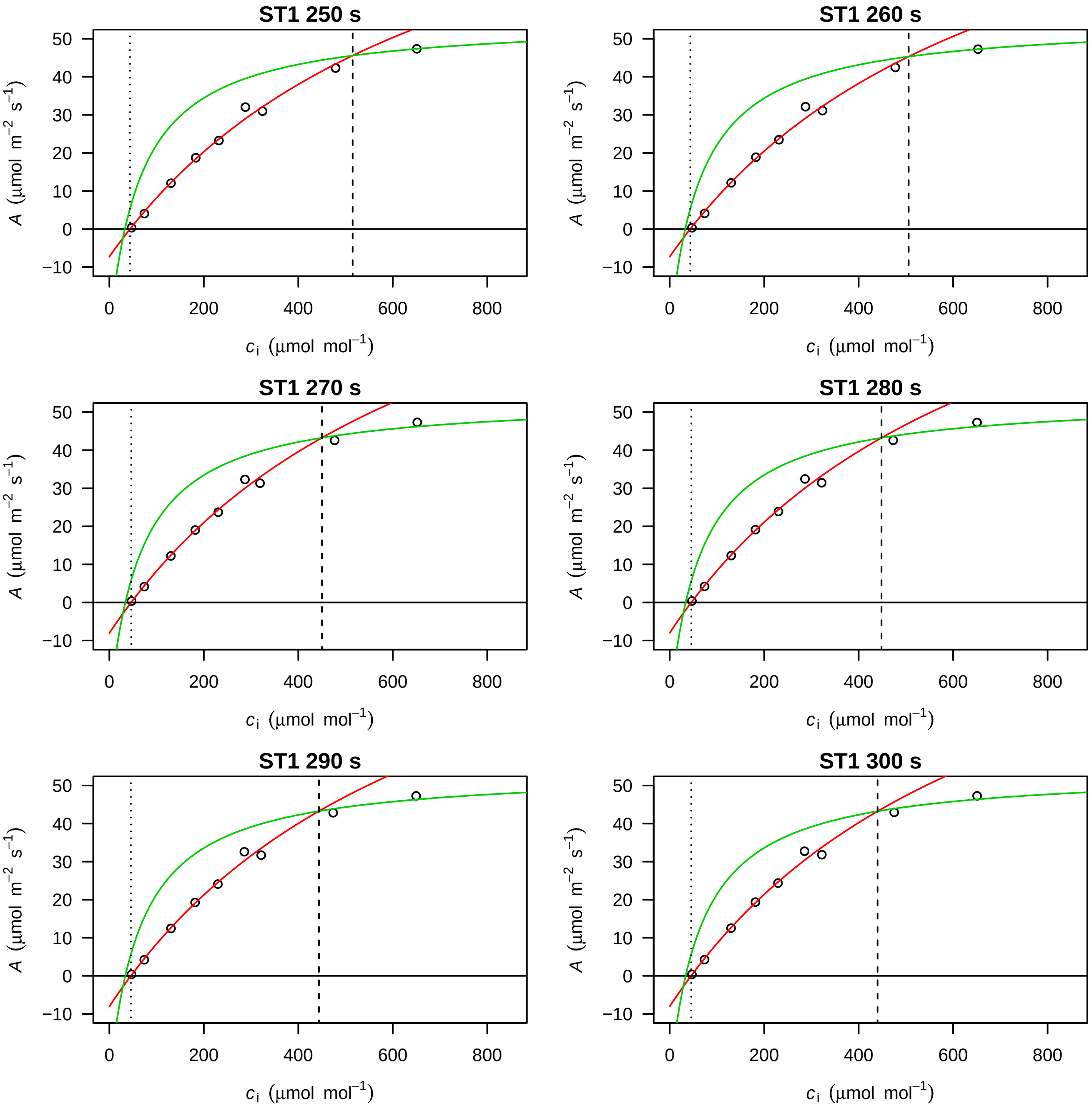
ST1 250 s
ST1 260 s
ST1 270 s
ST1 280 s
ST1 290 s
ST1 300 s
A (µmol m−2 s−1)
ci (µmol mol−1)
50
40
30
20
10
0
−10
0
200
400
600
800

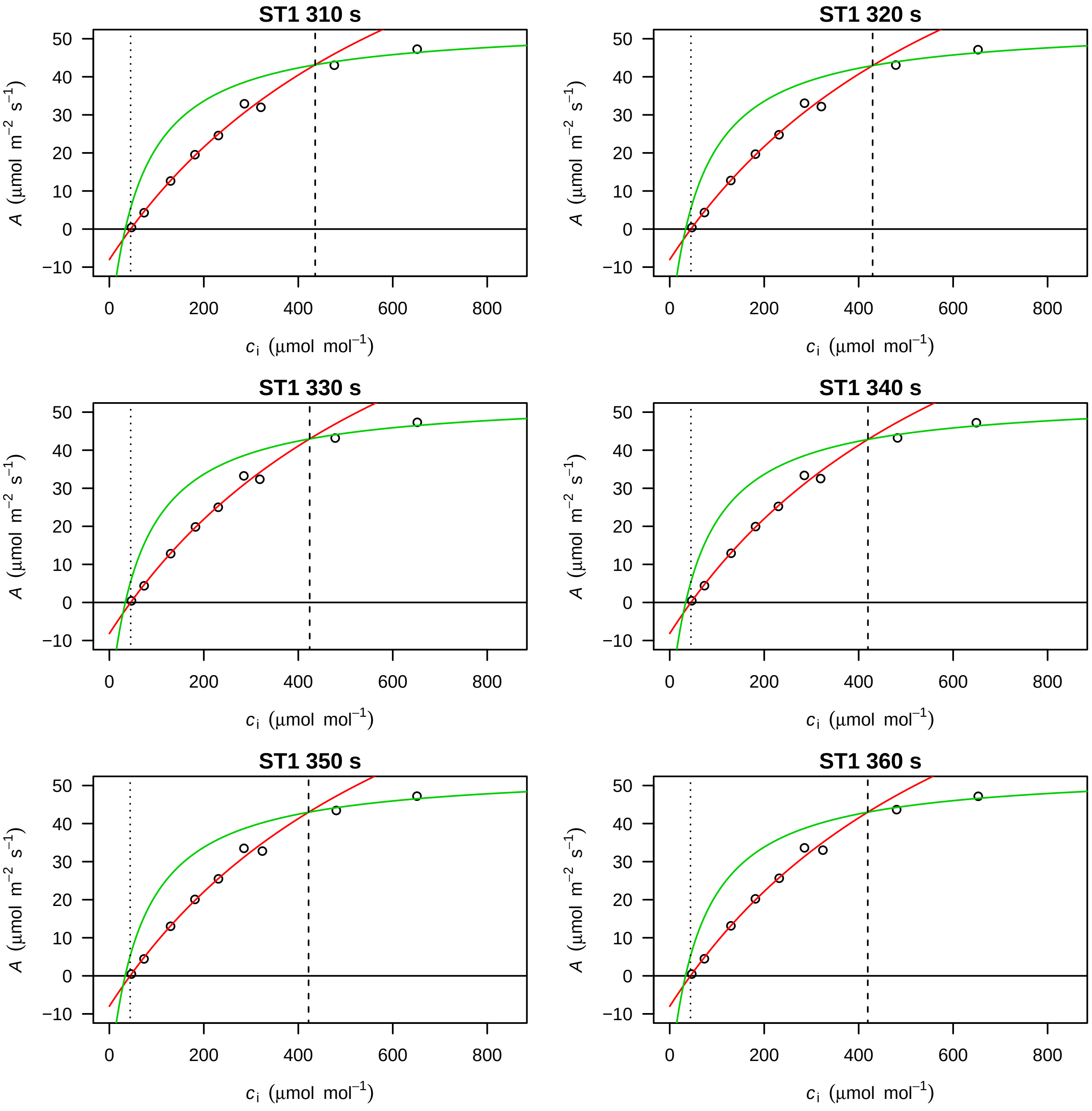

ST1 310 s
ST1 320 s
ST1 330 s
ST1 340 s
ST1 350 s
ST1 360 s
A (µmol m−2 s−1)
−10 0 10 20 30 40 50
0 200 400 600 800
ci (µmol mol−1)

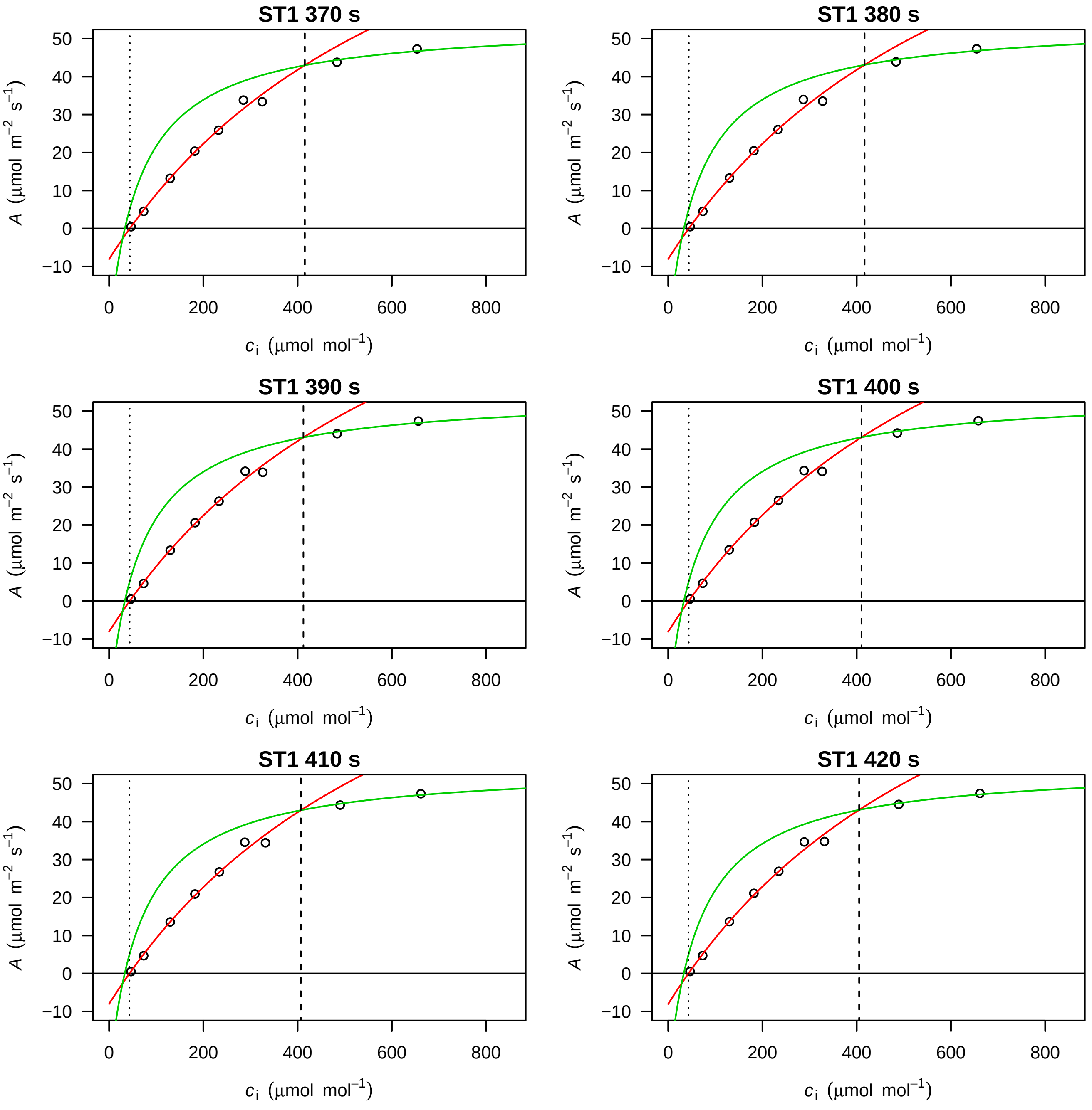
ST1 370 s
ST1 380 s
ST1 390 s
ST1 400 s
ST1 410 s
ST1 420 s
A (µmol m−2 s−1)
ci (µmol mol−1)
50
40
30
20
10
0
−10
0
200
400
600
800

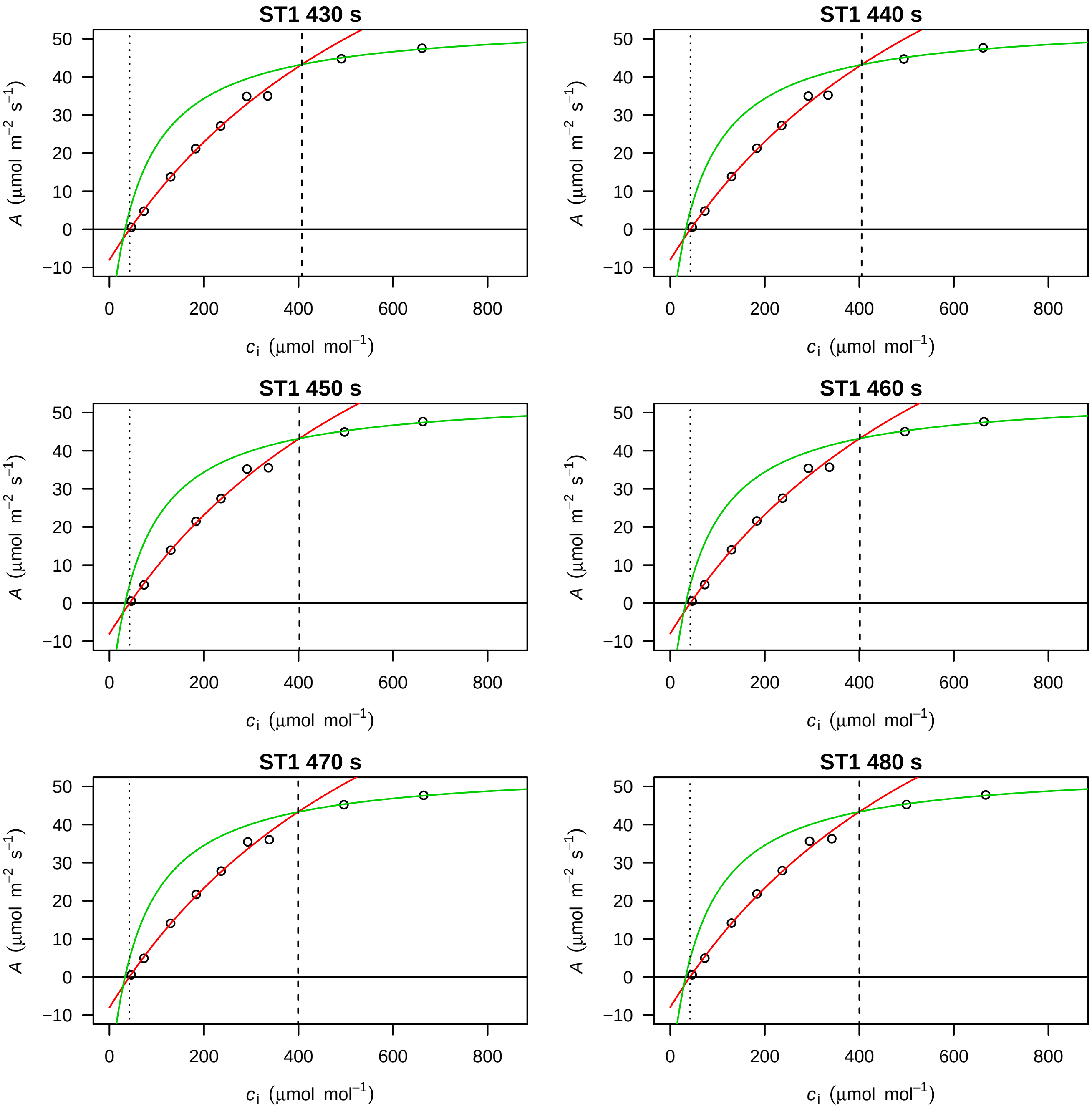

ST1 430 s
ST1 440 s
ST1 450 s
ST1 460 s
ST1 470 s
ST1 480 s
A (µmol m−2 s−1)
ci (µmol mol−1)
50
40
30
20
10
0
−10
0
200
400
600
800

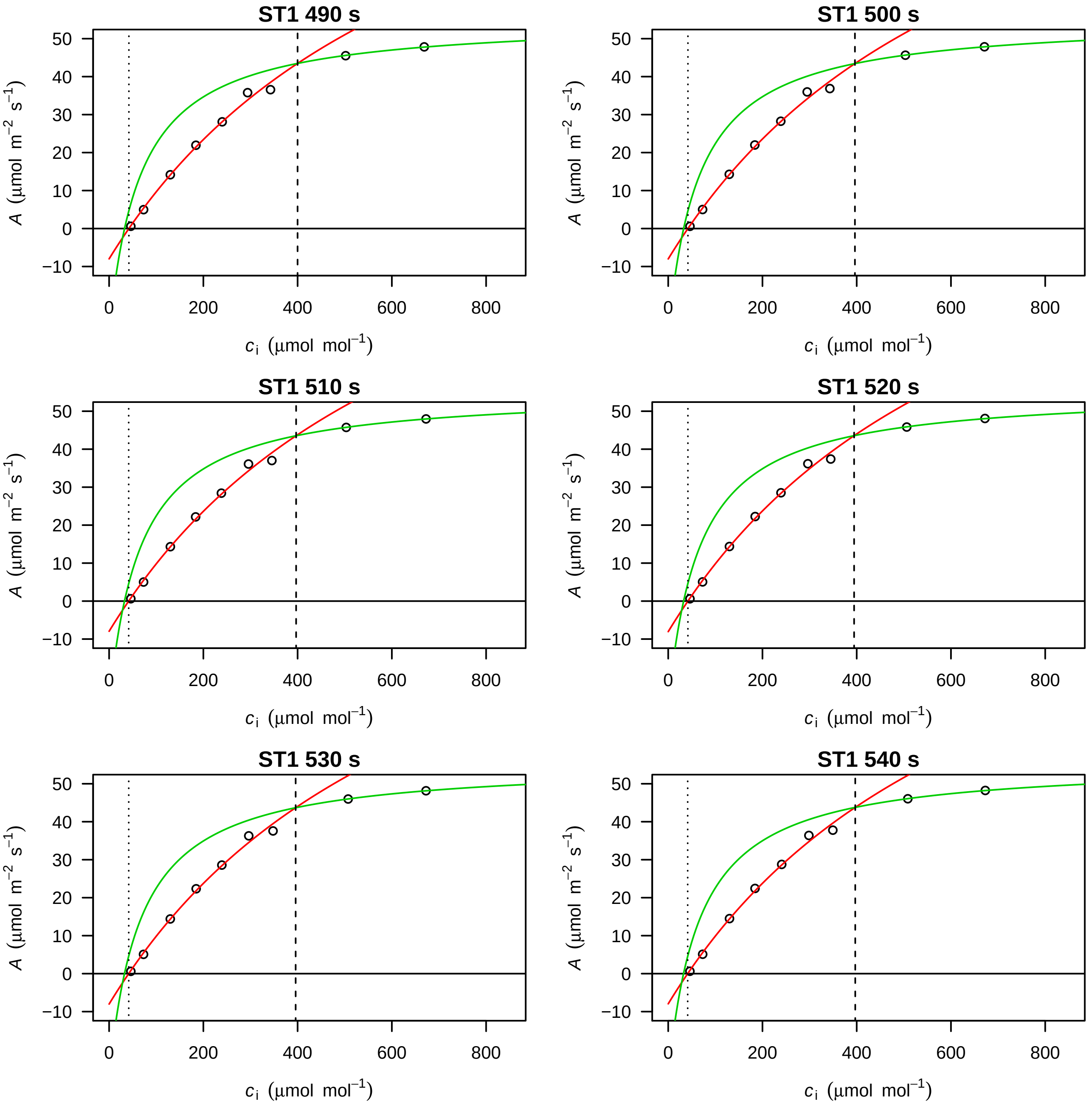
ST1 490 s
ST1 500 s
ST1 510 s
ST1 520 s
ST1 530 s
ST1 540 s
A (μmol m^{-2} s^{-1})
c_i (μmol mol^{-1})

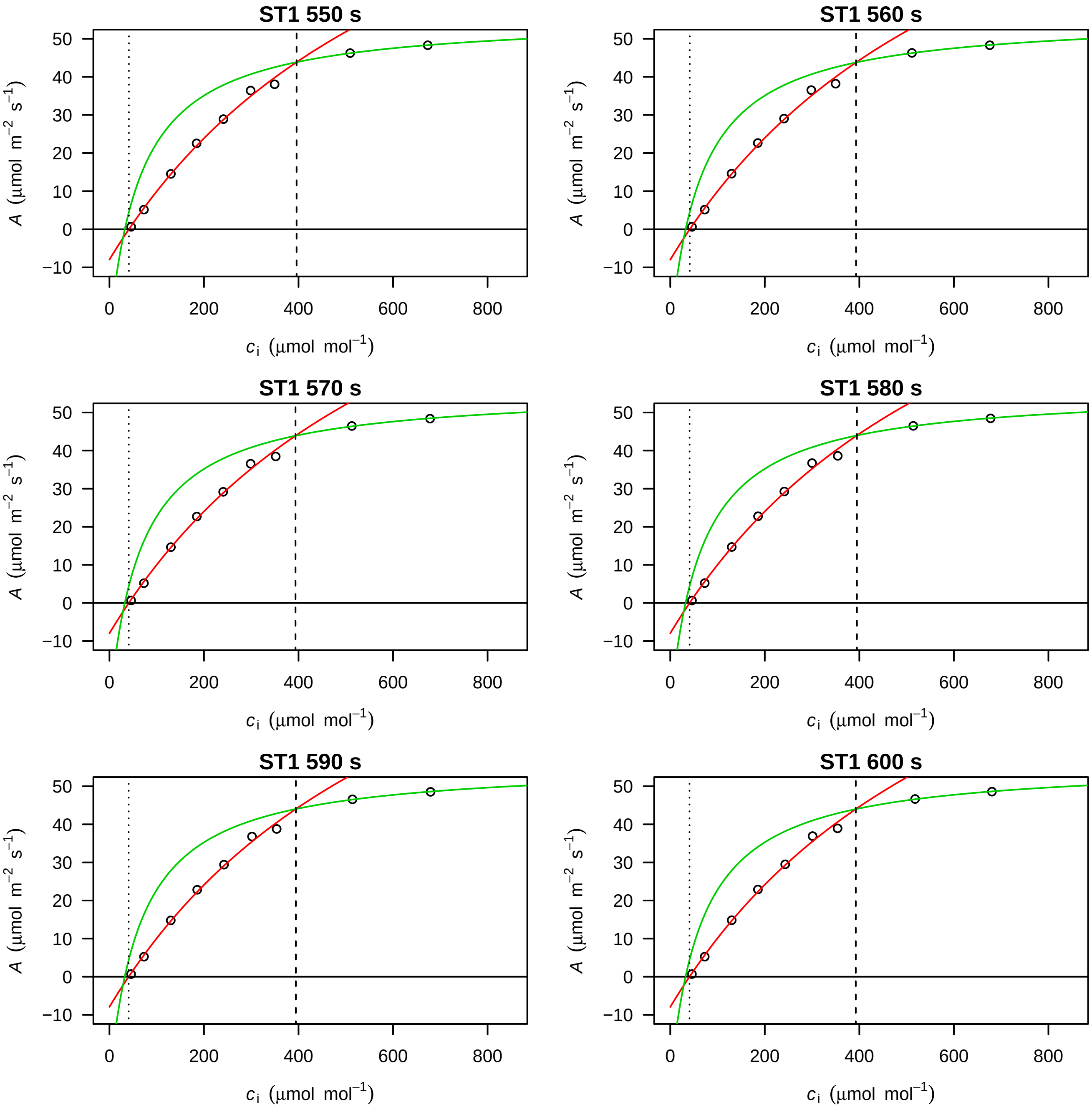
ST1 550 s
ST1 560 s
ST1 570 s
ST1 580 s
ST1 590 s
ST1 600 s
A (µmol m^{-2} s^{-1})
c_i (µmol mol^{-1})
50
40
30
20
10
0
−10
0
200
400
600
800

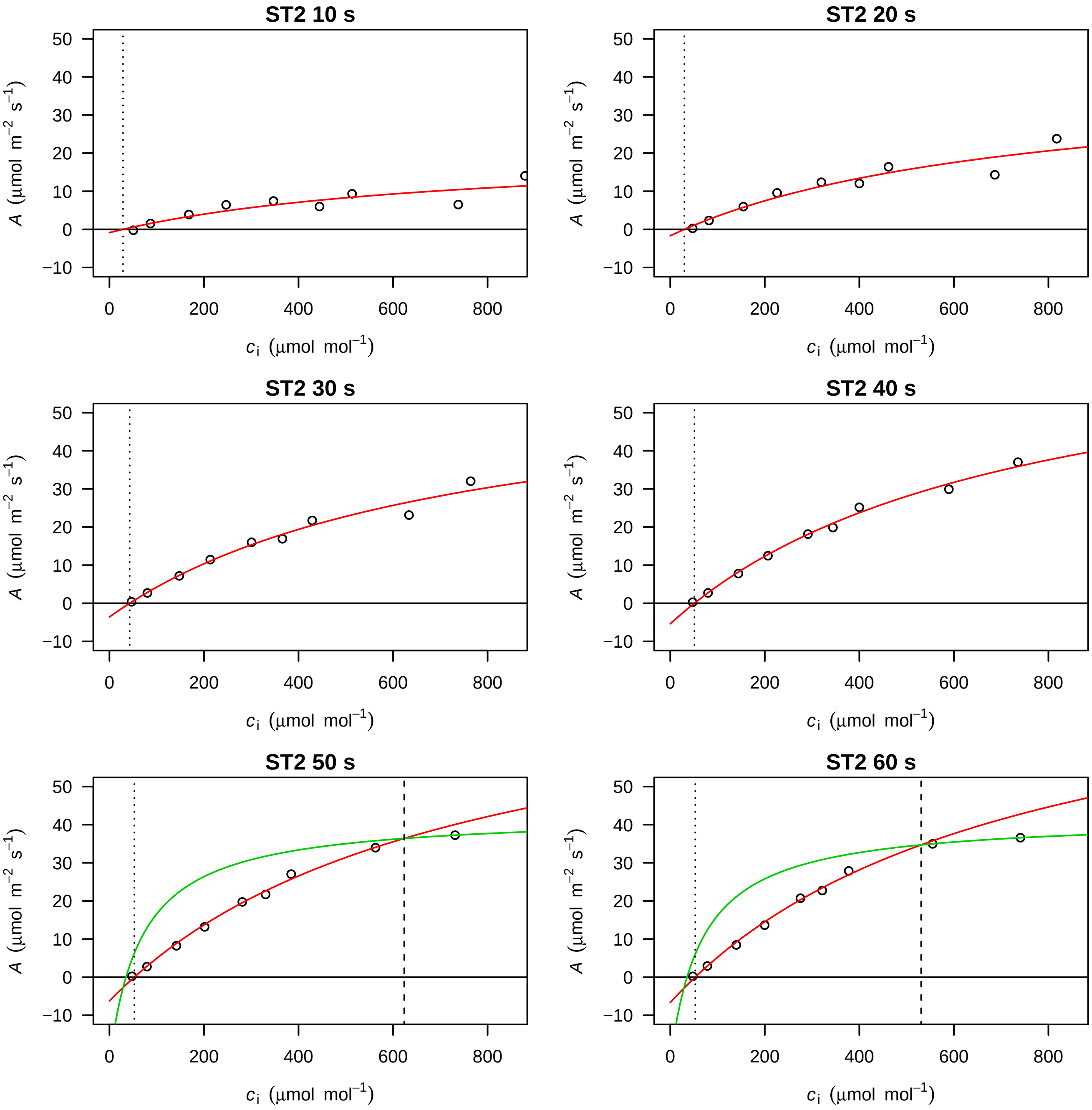
ST2 10 s
ST2 20 s
ST2 30 s
ST2 40 s
ST2 50 s
ST2 60 s
A (µmol m^{-2} s^{-1})
c_i (µmol mol^{-1})

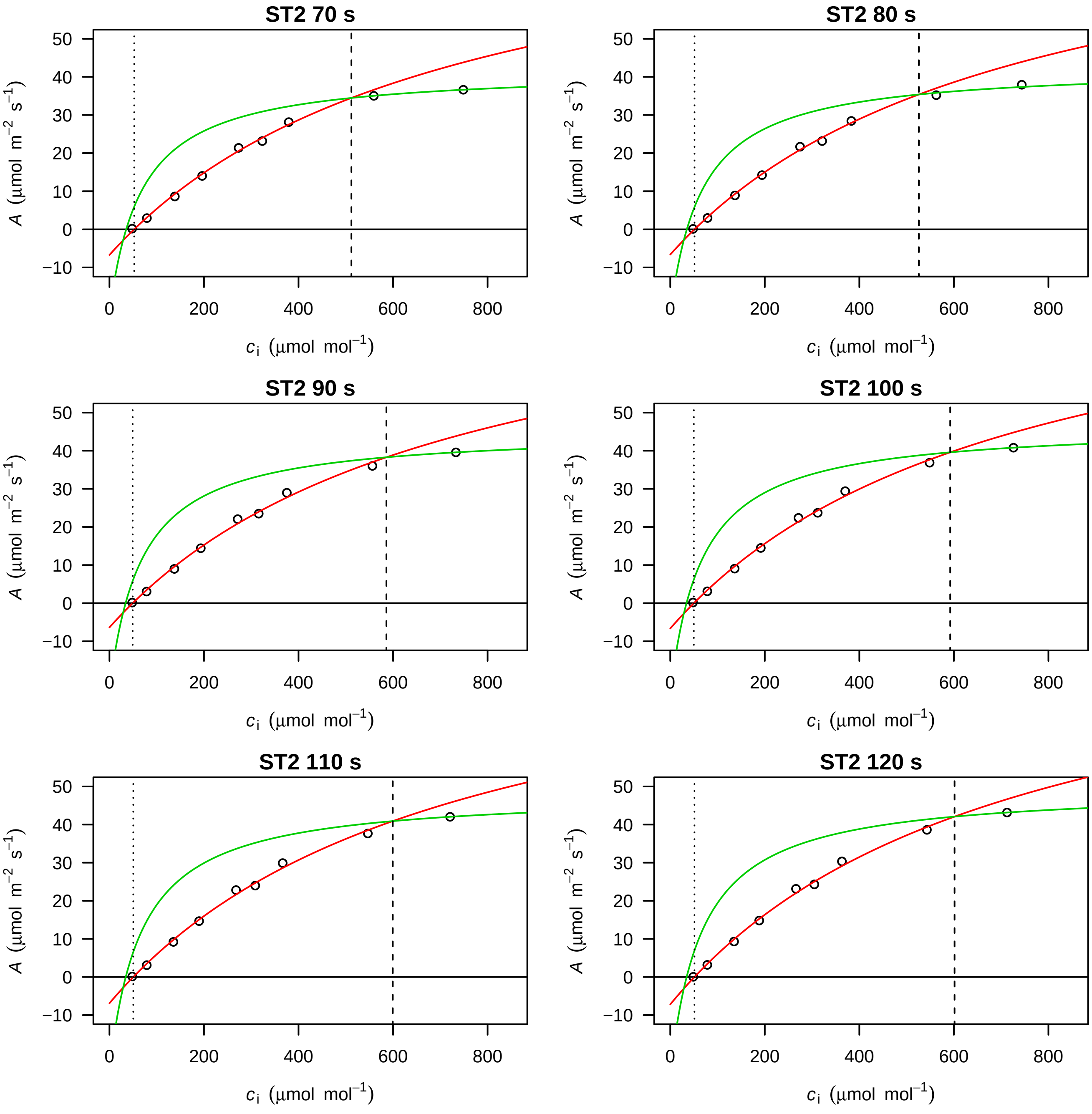
ST2 70 s
ST2 80 s
ST2 90 s
ST2 100 s
ST2 110 s
ST2 120 s
A (µmol m−2 s−1)
ci (µmol mol−1)
50
40
30
20
10
0
−10
0
200
400
600
800

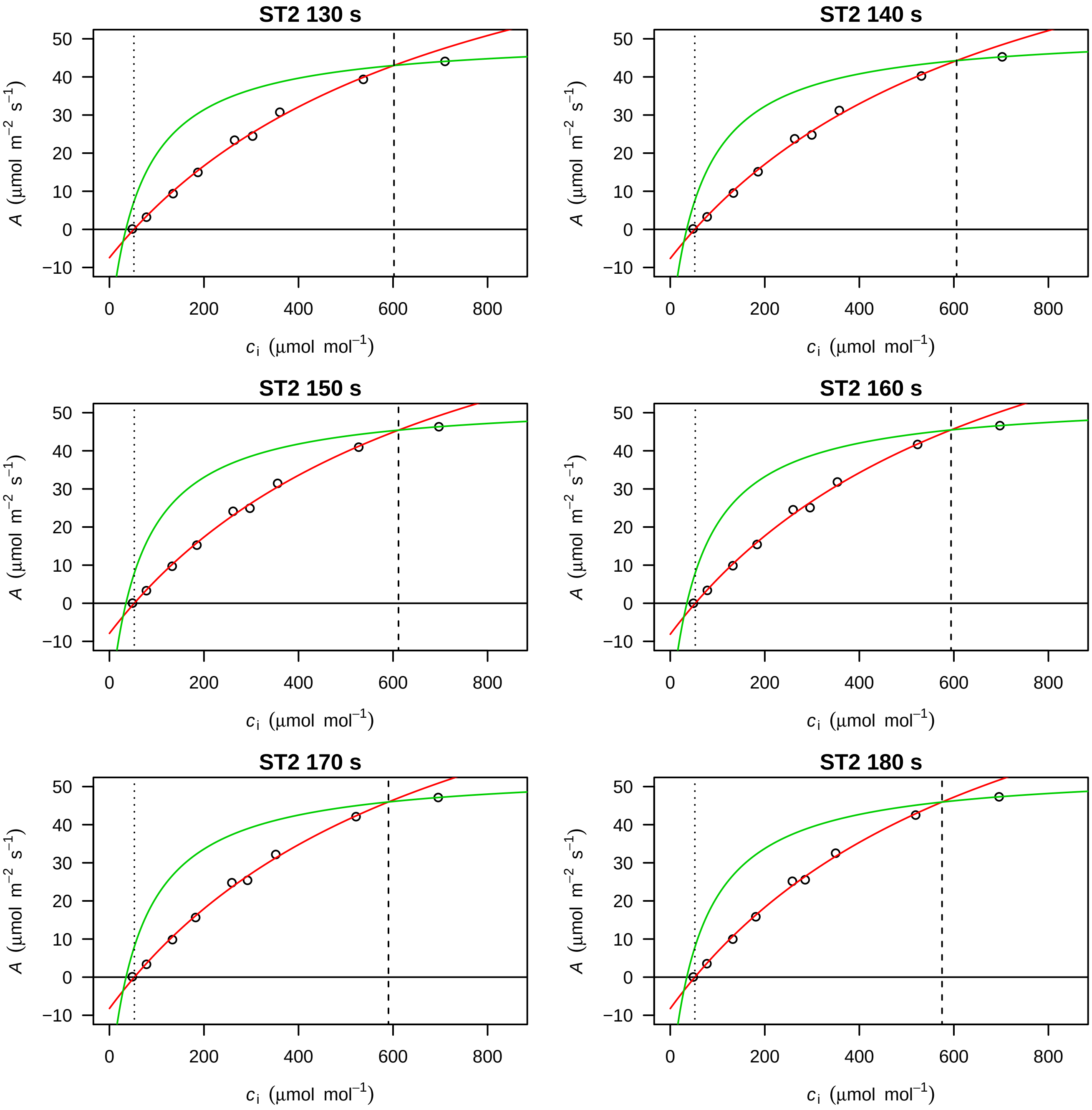

ST2 130 s
ST2 140 s
ST2 150 s
ST2 160 s
ST2 170 s
ST2 180 s
A (μmol m−2 s−1)
ci (μmol mol−1)

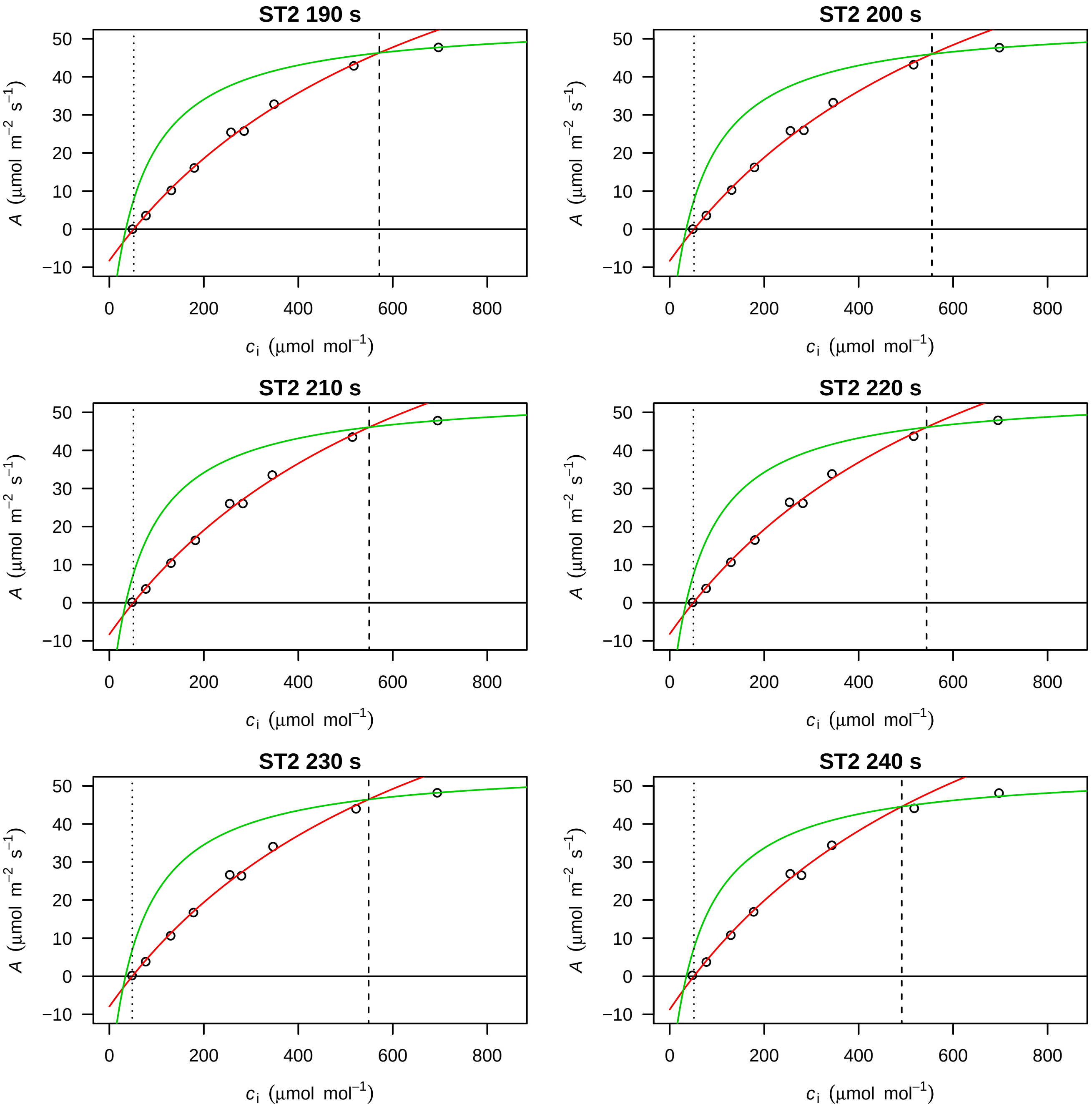
ST2 190 s
ST2 200 s
ST2 210 s
ST2 220 s
ST2 230 s
ST2 240 s
A (μmol m^{-2} s^{-1})
c_i (μmol mol^{-1})
50
40
30
20
10
0
−10
0
200
400
600
800

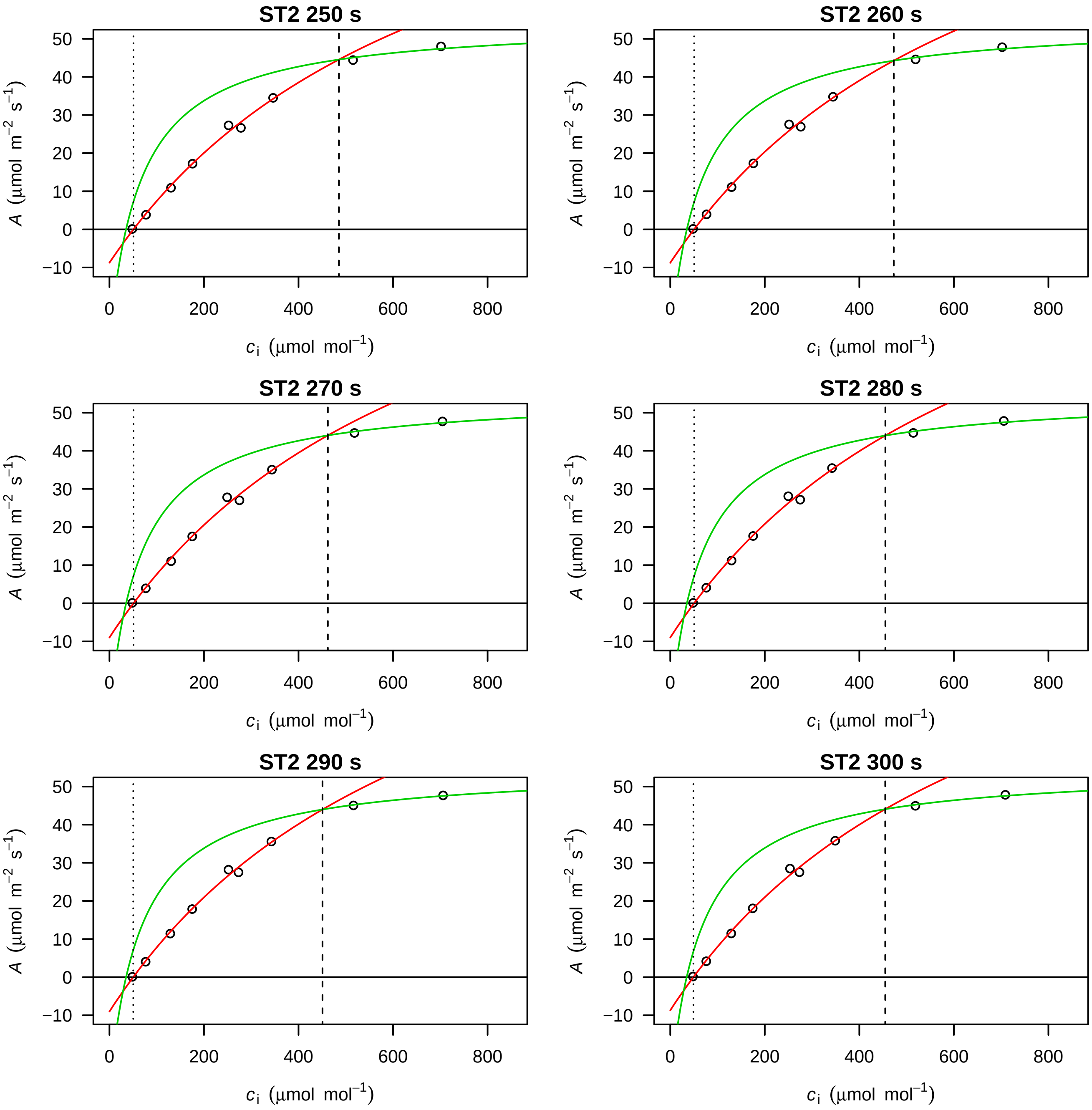
ST2 250 s
ST2 260 s
ST2 270 s
ST2 280 s
ST2 290 s
ST2 300 s
A (µmol m−2 s−1)
ci (µmol mol−1)
−10
0
10
20
30
40
50
0
200
400
600
800

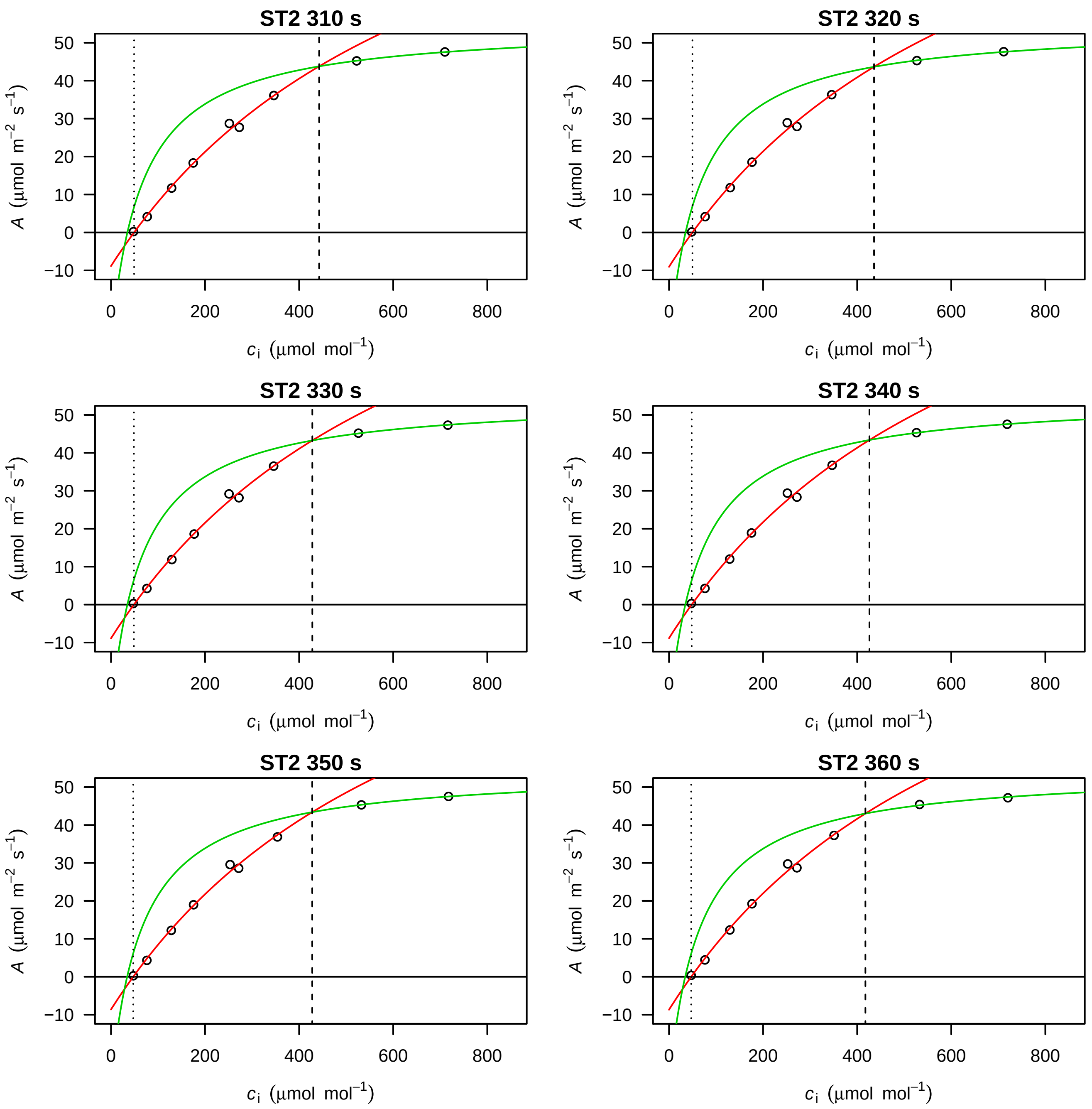

ST2 310 s
ST2 320 s
ST2 330 s
ST2 340 s
ST2 350 s
ST2 360 s
A (µmol m^{-2} s^{-1})
c_i (µmol mol^{-1})

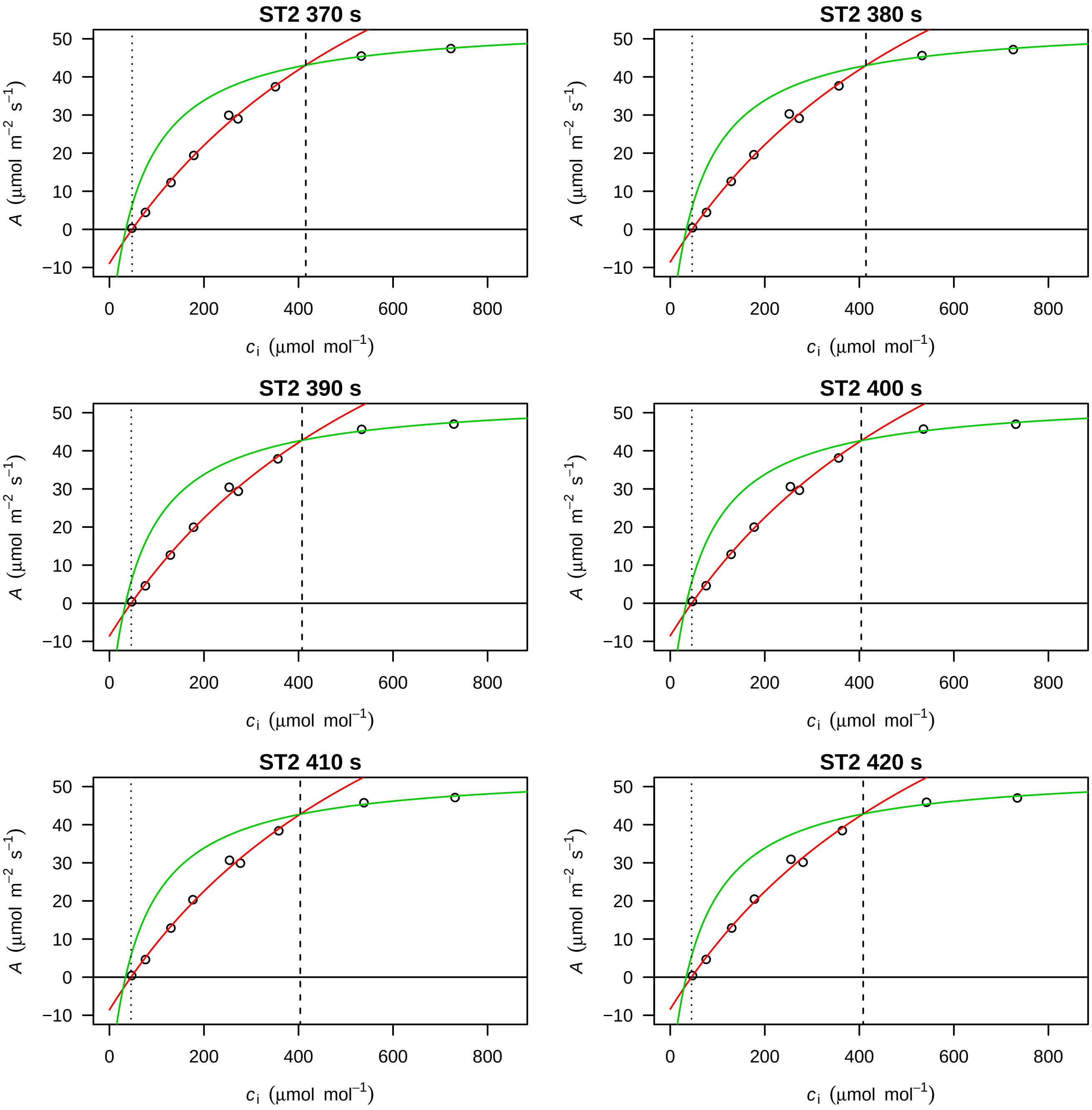

ST2 370 s
ST2 380 s
ST2 390 s
ST2 400 s
ST2 410 s
ST2 420 s
A (µmol m−2 s−1)
ci (µmol mol−1)
−10
0
10
20
30
40
50
0
200
400
600
800

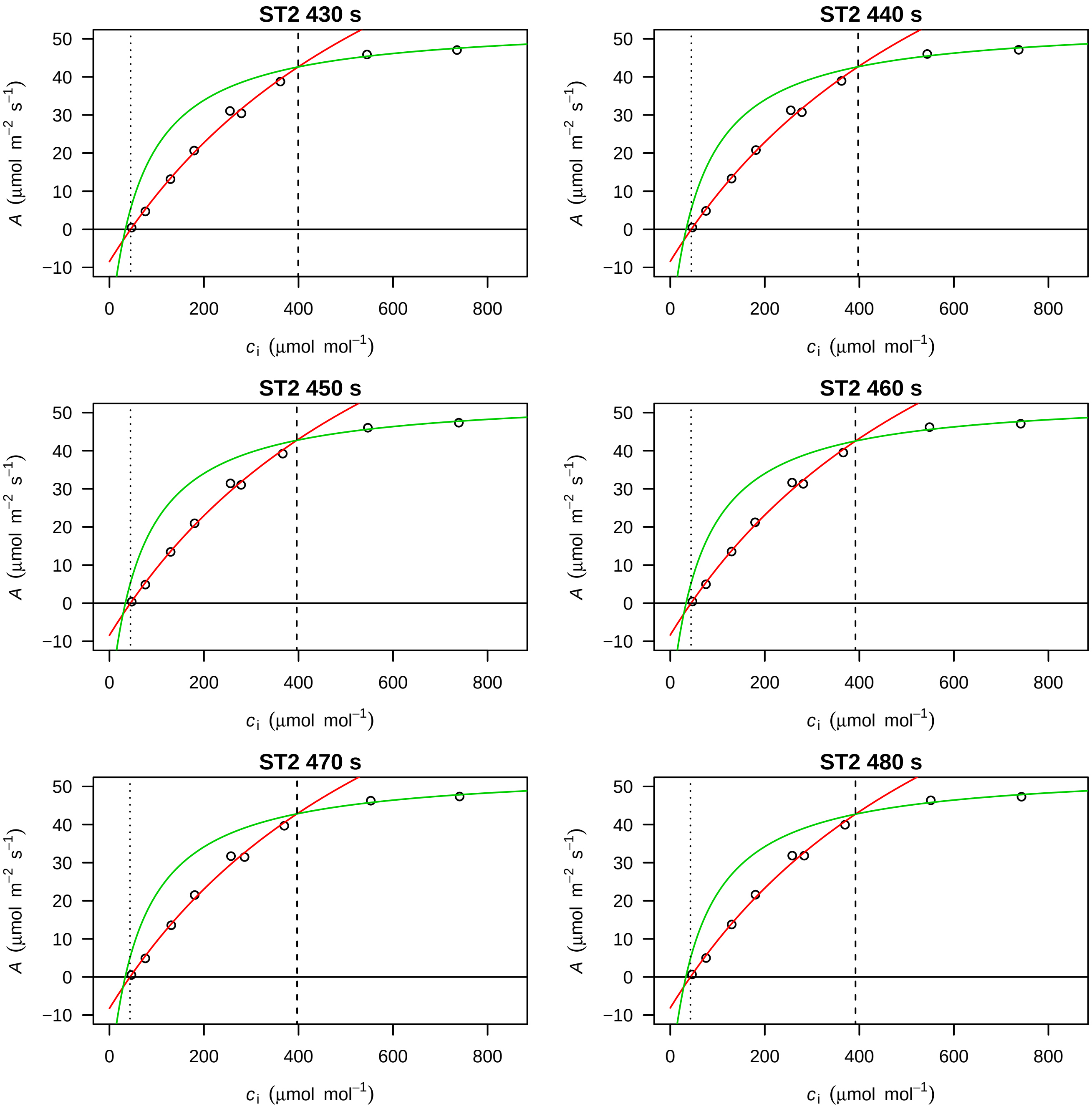

ST2 430 s
ST2 440 s
ST2 450 s
ST2 460 s
ST2 470 s
ST2 480 s
A (µmol m−2 s−1)
ci (µmol mol−1)
−10
0
10
20
30
40
50
0
200
400
600
800

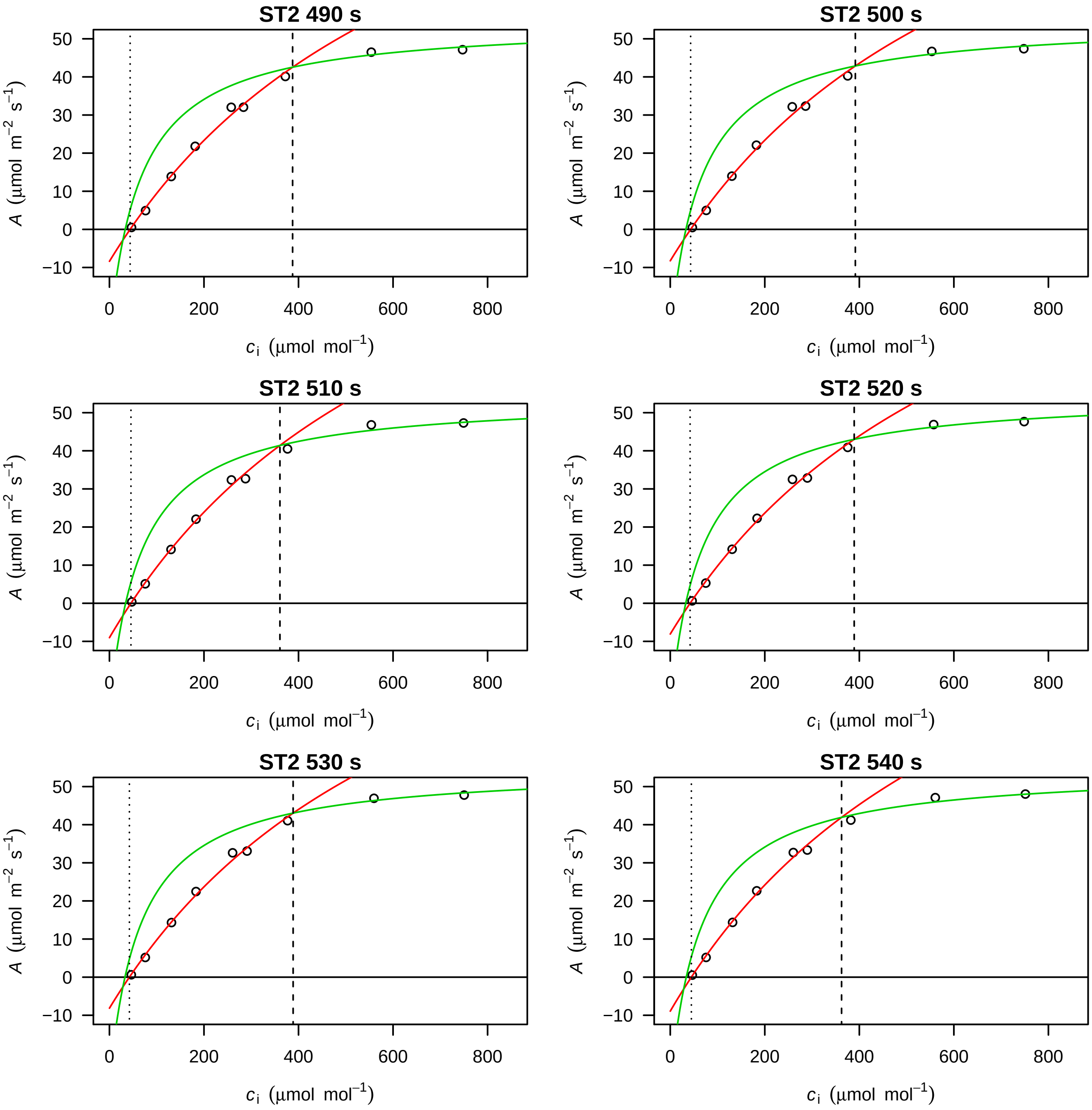
ST2 490 s
ST2 500 s
ST2 510 s
ST2 520 s
ST2 530 s
ST2 540 s
A (μmol m−2 s−1)
ci (μmol mol−1)
50
40
30
20
10
0
−10
0
200
400
600
800

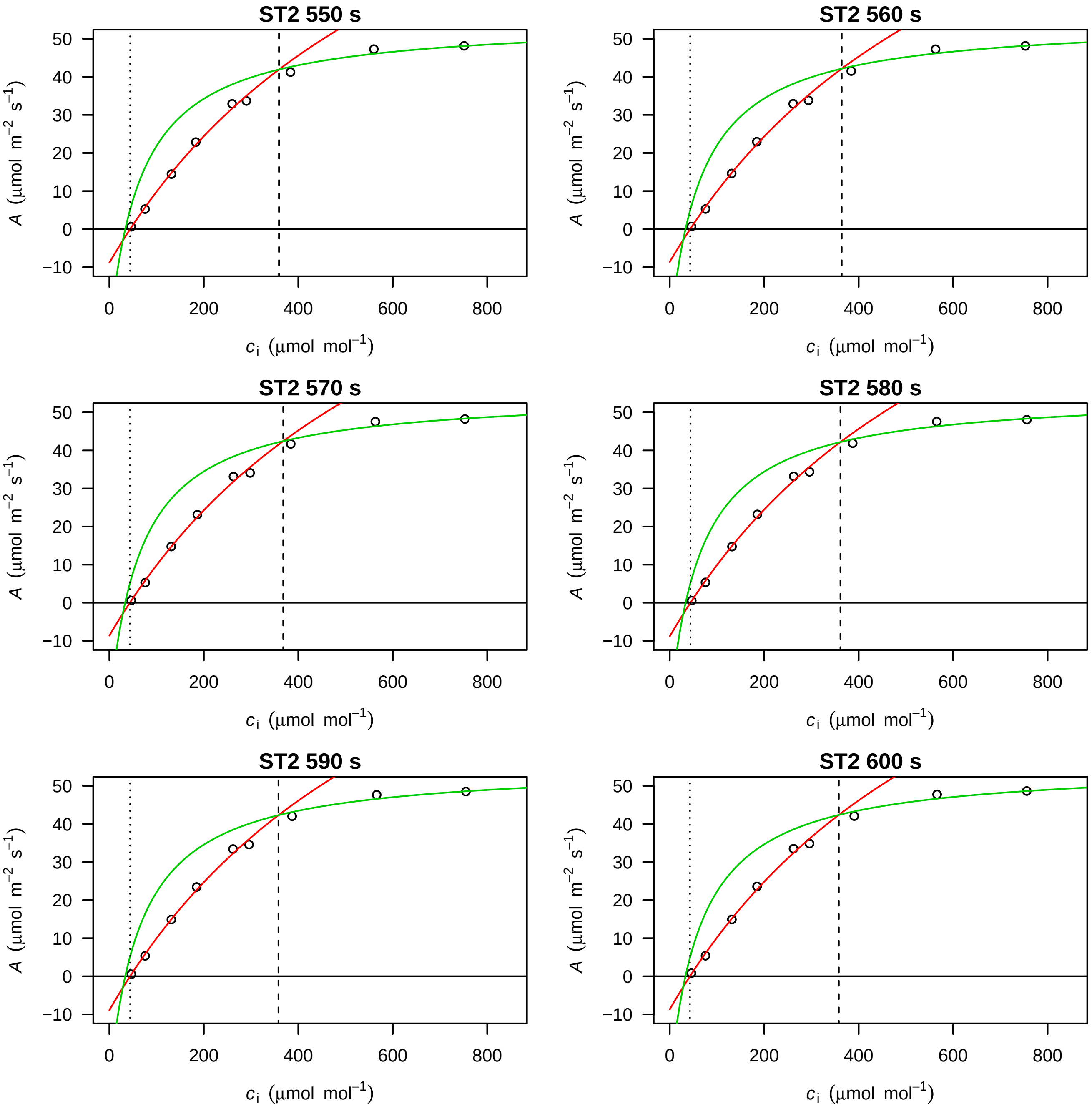
ST2 550 s
ST2 560 s
ST2 570 s
ST2 580 s
ST2 590 s
ST2 600 s
A (µmol m−2 s−1)
ci (µmol mol−1)
50
40
30
20
10
0
−10
0
200
400
600
800

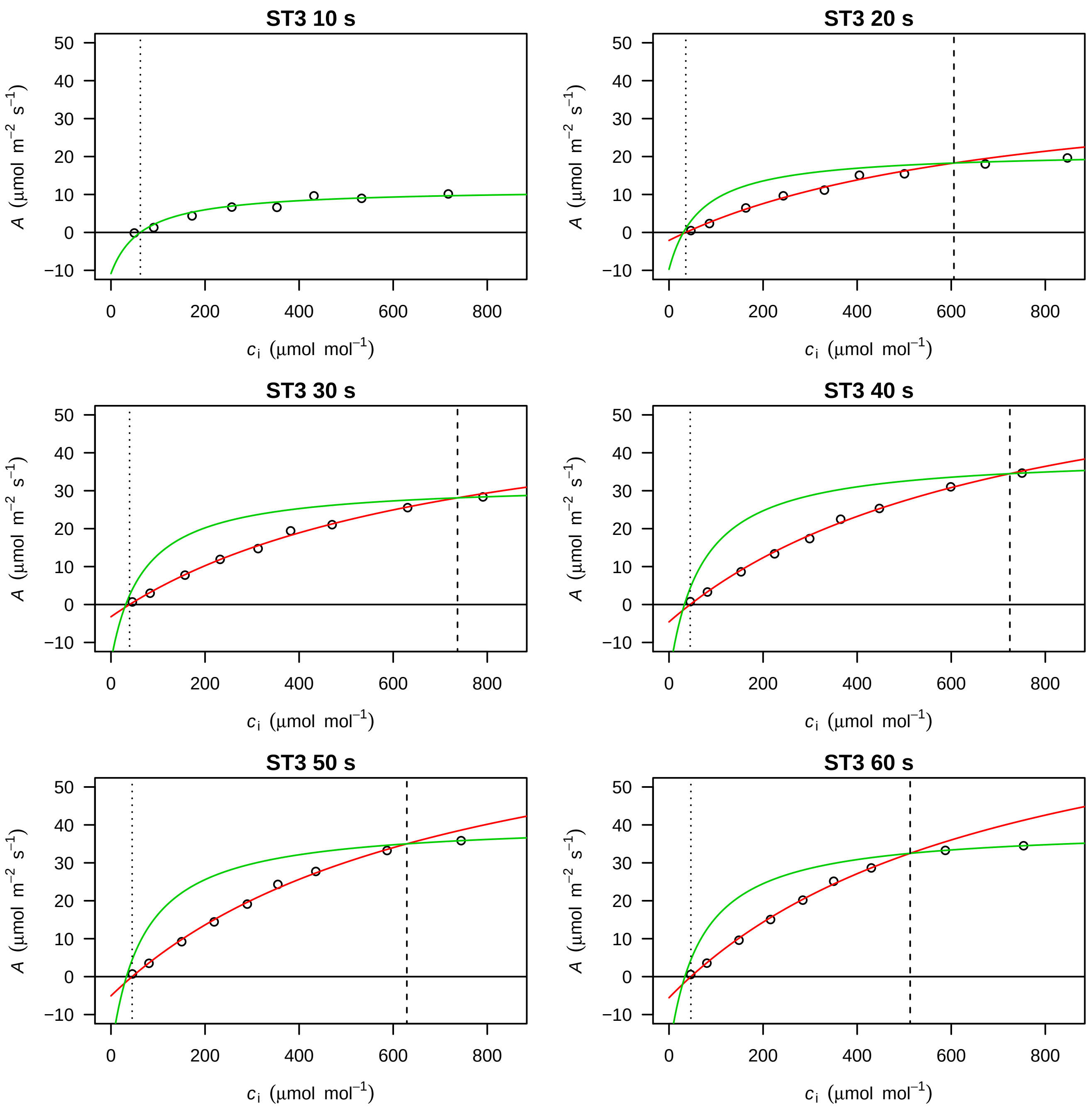

ST3 10 s
ST3 20 s
ST3 30 s
ST3 40 s
ST3 50 s
ST3 60 s
A (µmol m−2 s−1)
ci (µmol mol−1)
50
40
30
20
10
0
−10
0
200
400
600
800

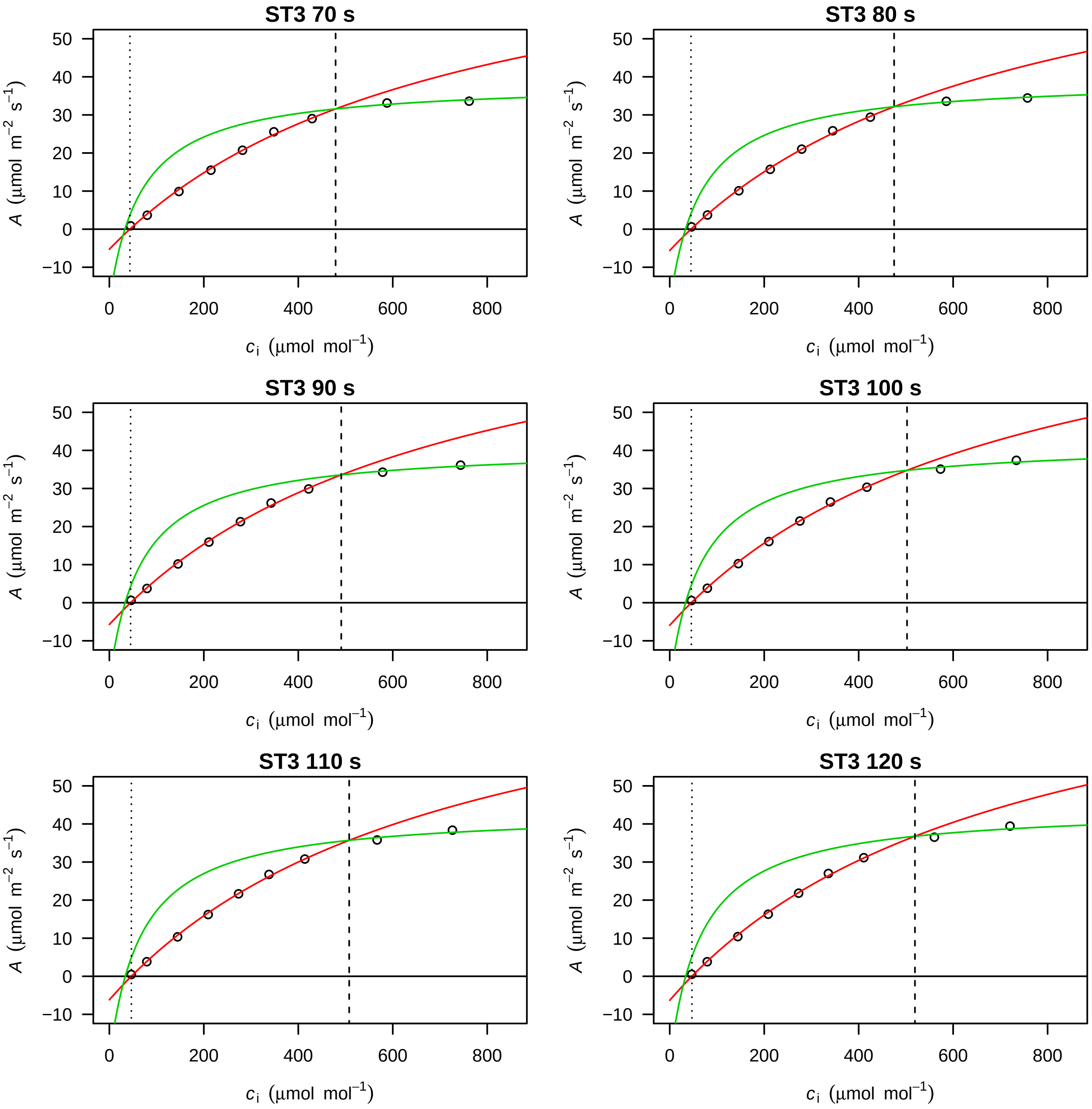
ST3 70 s
ST3 80 s
ST3 90 s
ST3 100 s
ST3 110 s
ST3 120 s
A (µmol m^{-2} s^{-1})
c_i (µmol mol^{-1})
50
40
30
20
10
0
−10
0
200
400
600
800

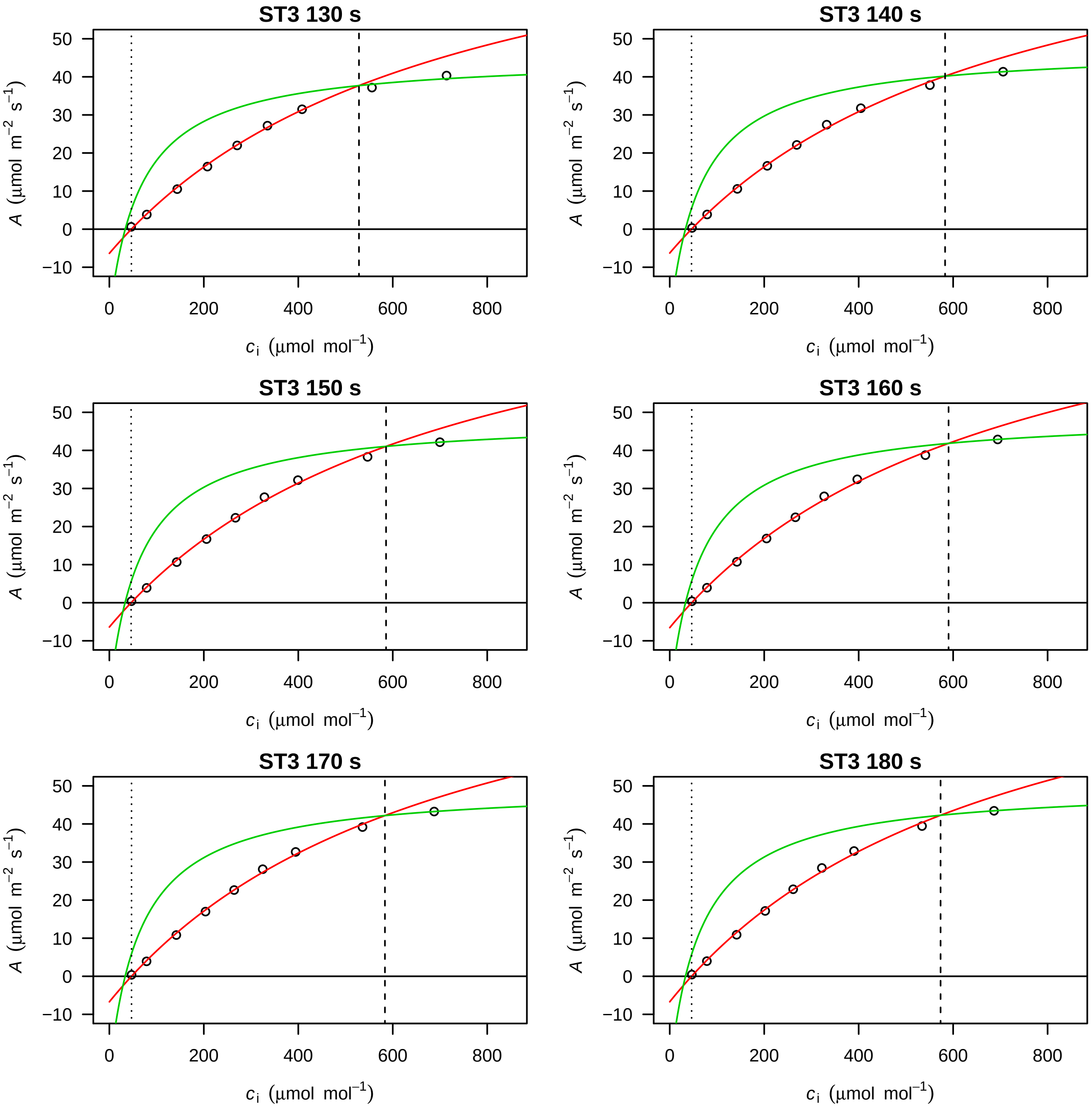
ST3 130 s
ST3 140 s
ST3 150 s
ST3 160 s
ST3 170 s
ST3 180 s
A (µmol m^{-2} s^{-1})
c_i (µmol mol^{-1})
50
40
30
20
10
0
−10
0
200
400
600
800

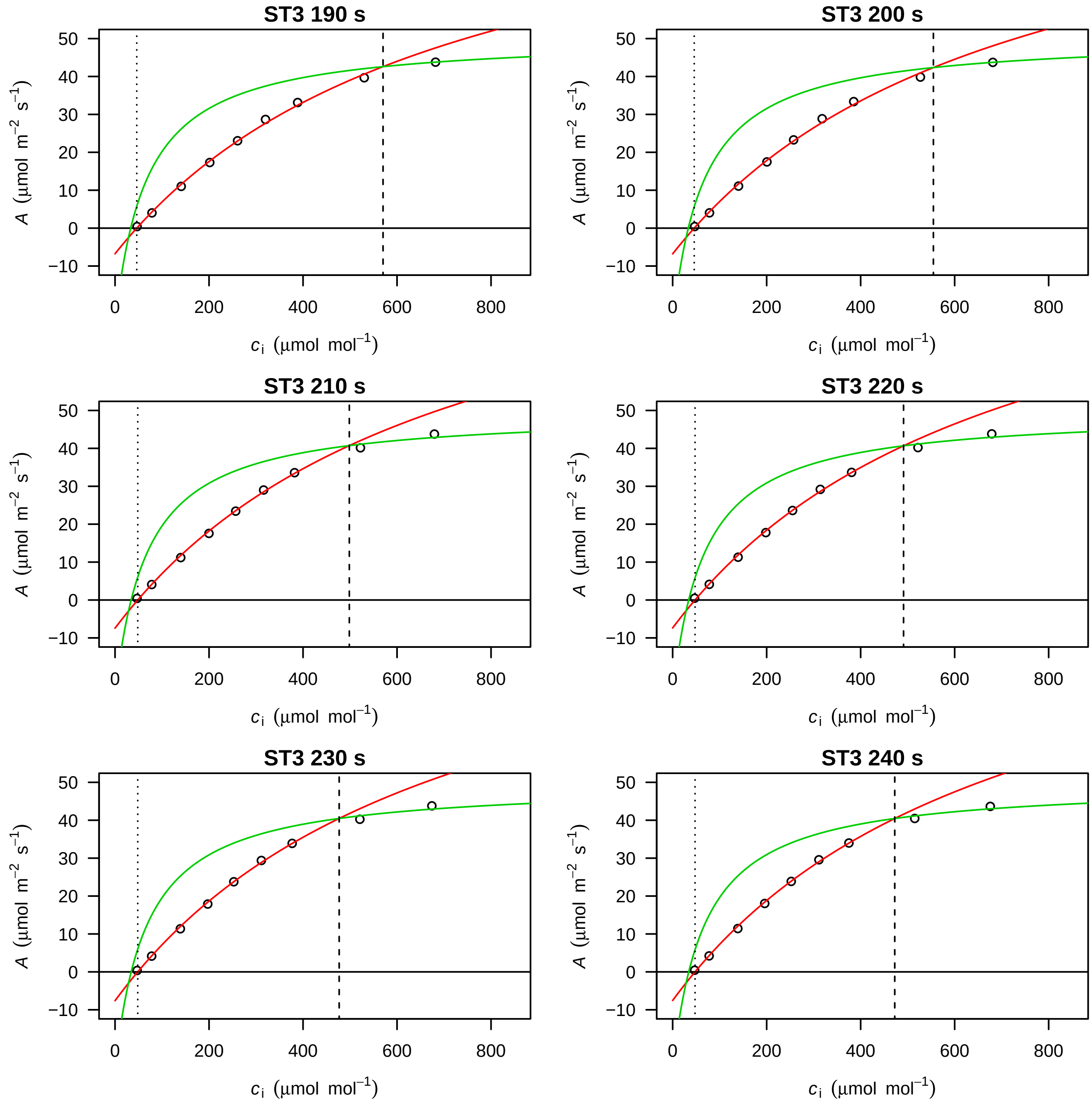
ST3 190 s
ST3 200 s
ST3 210 s
ST3 220 s
ST3 230 s
ST3 240 s
A (µmol m−2 s−1)
ci (µmol mol−1)
50
40
30
20
10
0
−10
0
200
400
600
800

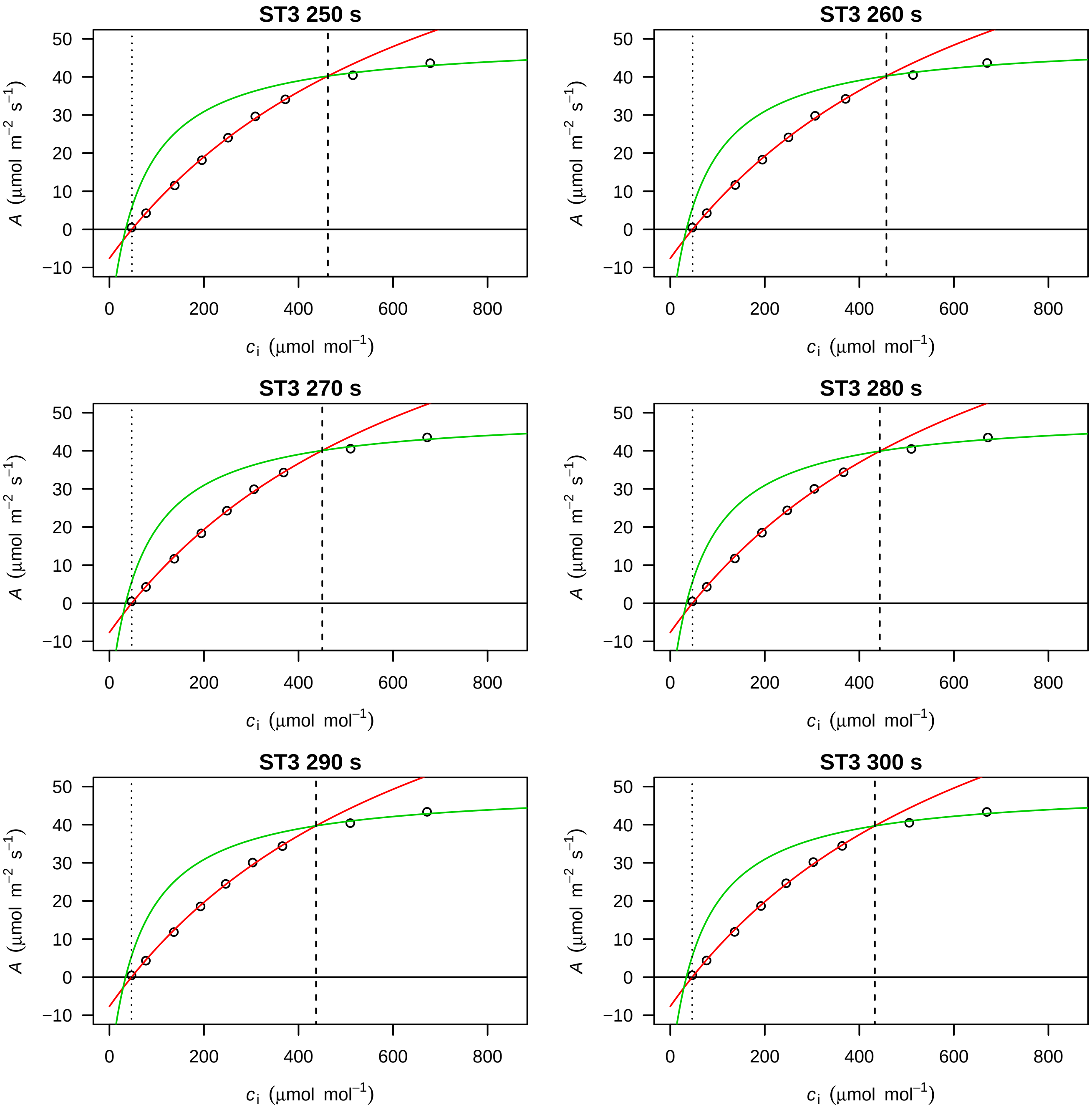

ST3 250 s
ST3 260 s
ST3 270 s
ST3 280 s
ST3 290 s
ST3 300 s
A (µmol m−2 s−1)
ci (µmol mol−1)
50
40
30
20
10
0
−10
0
200
400
600
800

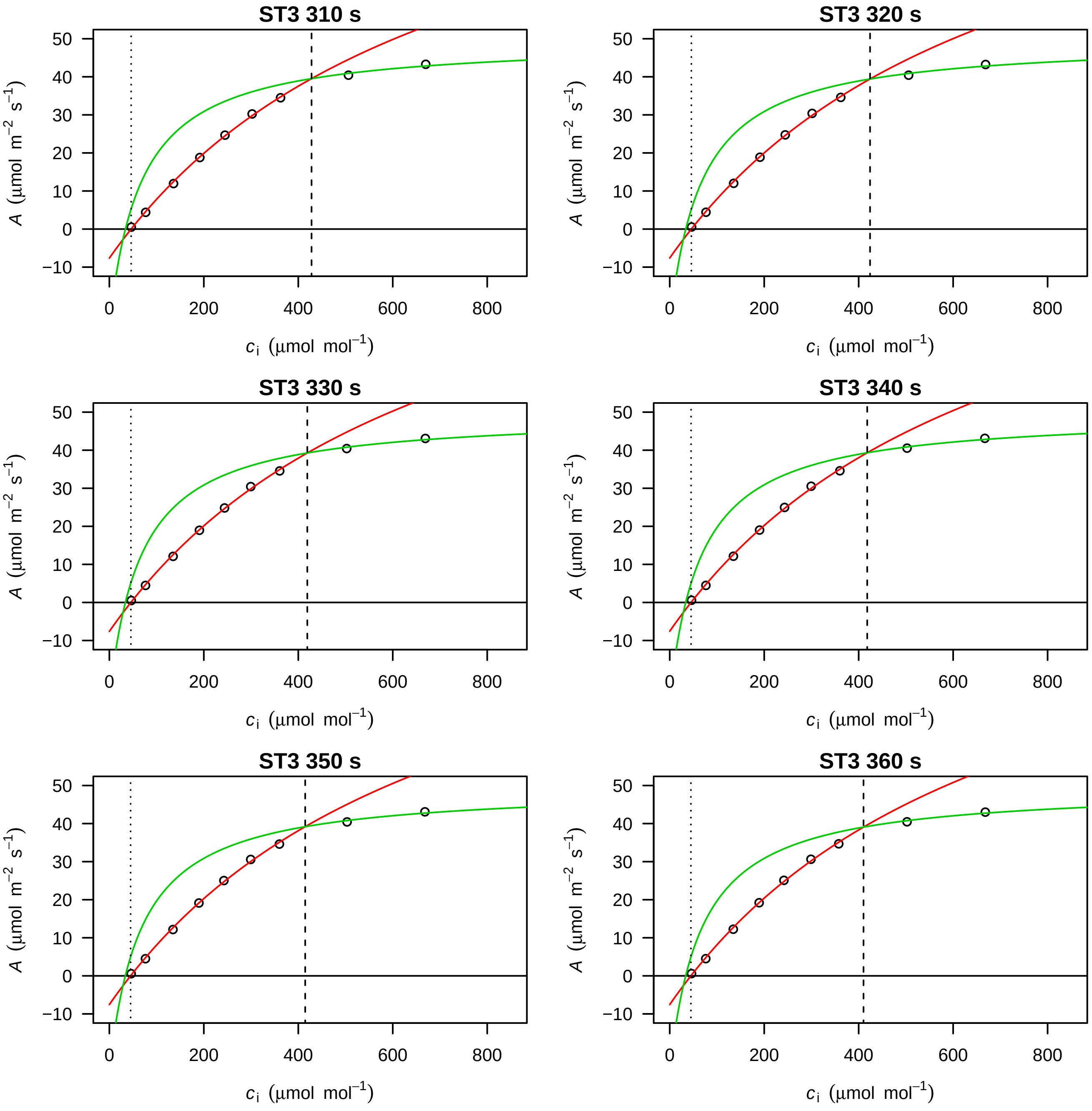
ST3 310 s
ST3 320 s
ST3 330 s
ST3 340 s
ST3 350 s
ST3 360 s
A (µmol m−2 s−1)
ci (µmol mol−1)
50
40
30
20
10
0
−10
0
200
400
600
800

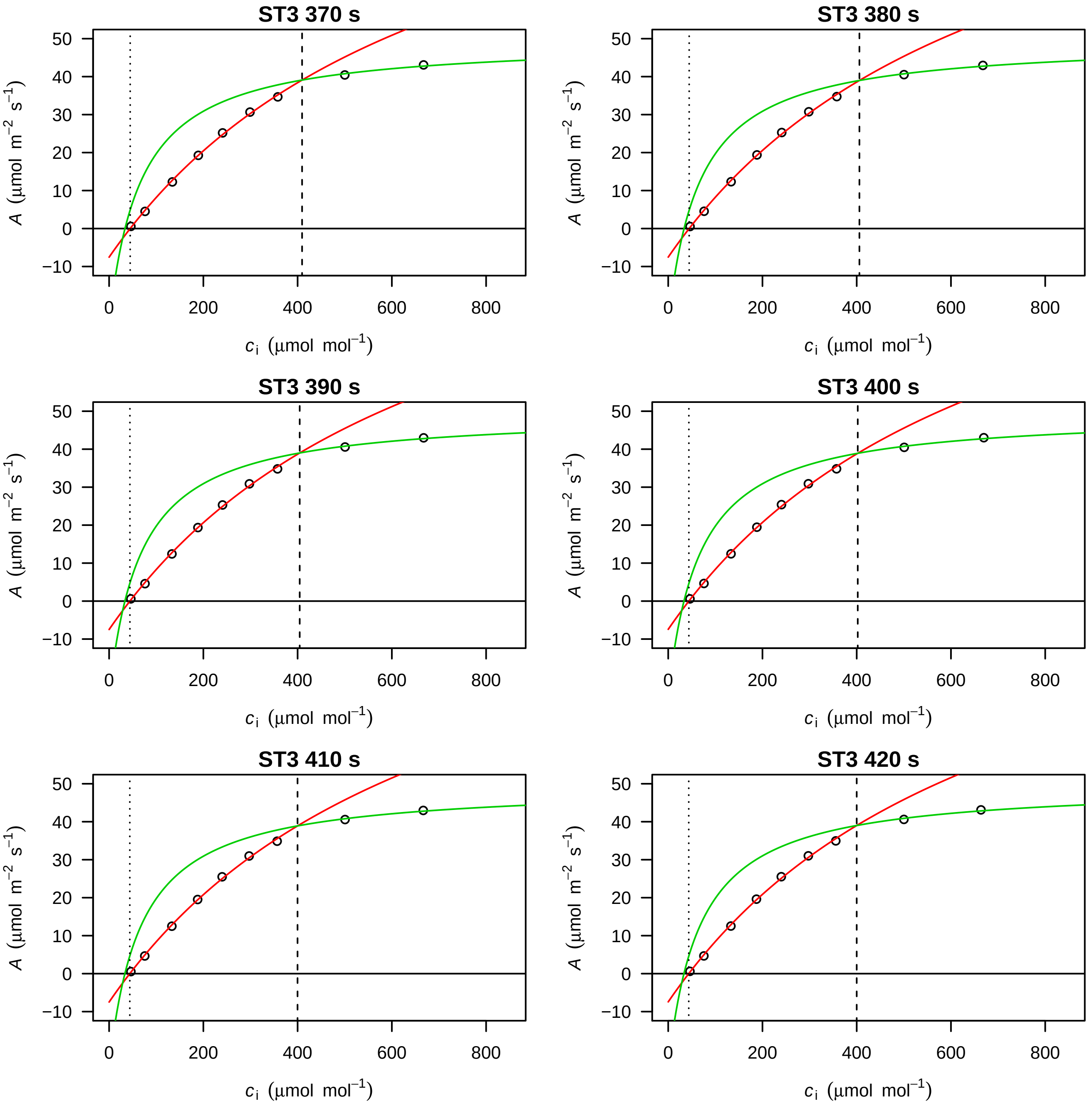
ST3 370 s
ST3 380 s
ST3 390 s
ST3 400 s
ST3 410 s
ST3 420 s
A (µmol m−2 s−1)
ci (µmol mol−1)
50
40
30
20
10
0
−10
0
200
400
600
800

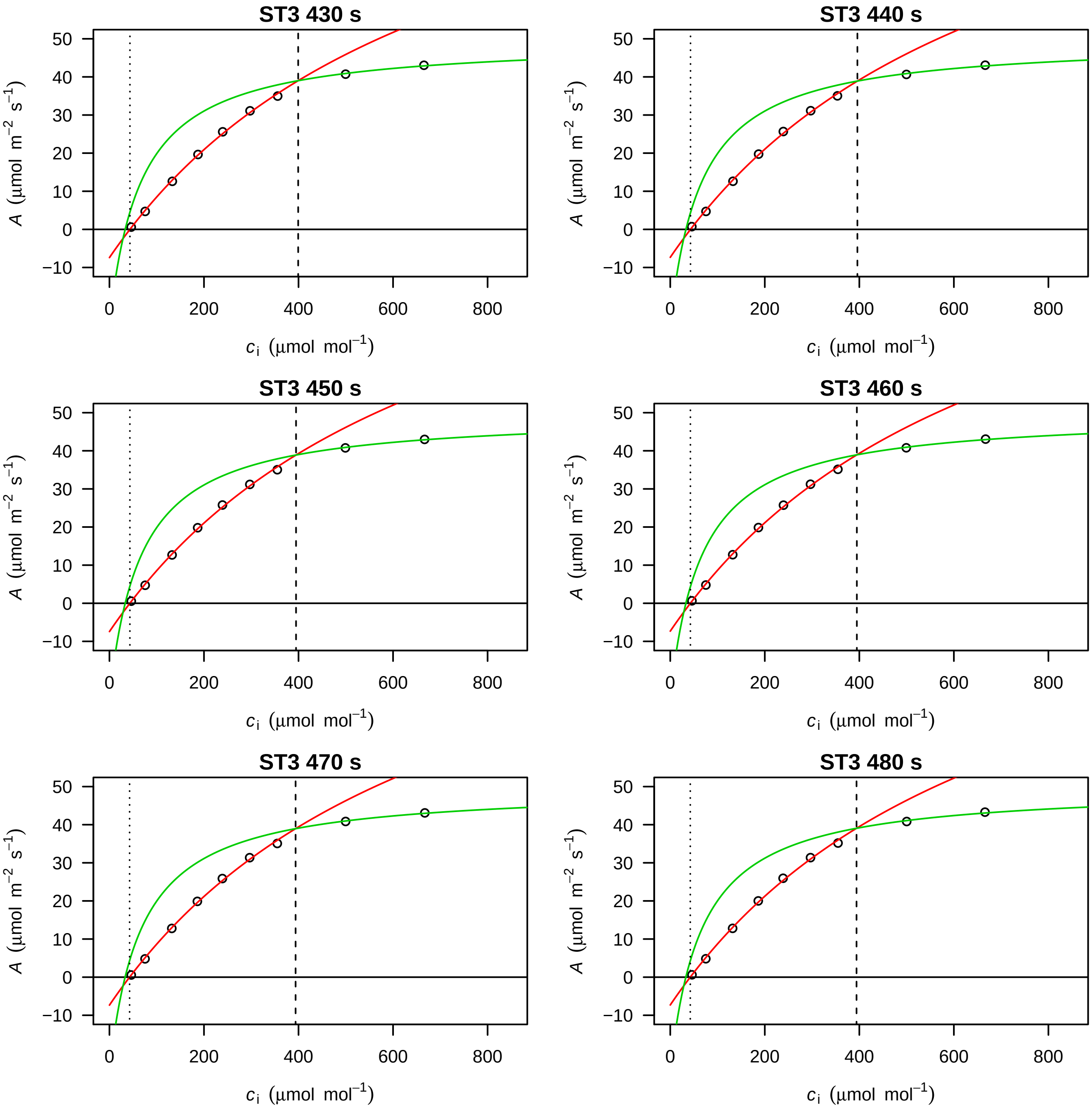

ST3 430 s
ST3 440 s
ST3 450 s
ST3 460 s
ST3 470 s
ST3 480 s
A (µmol m−2 s−1)
ci (µmol mol−1)
50
40
30
20
10
0
−10
0
200
400
600
800

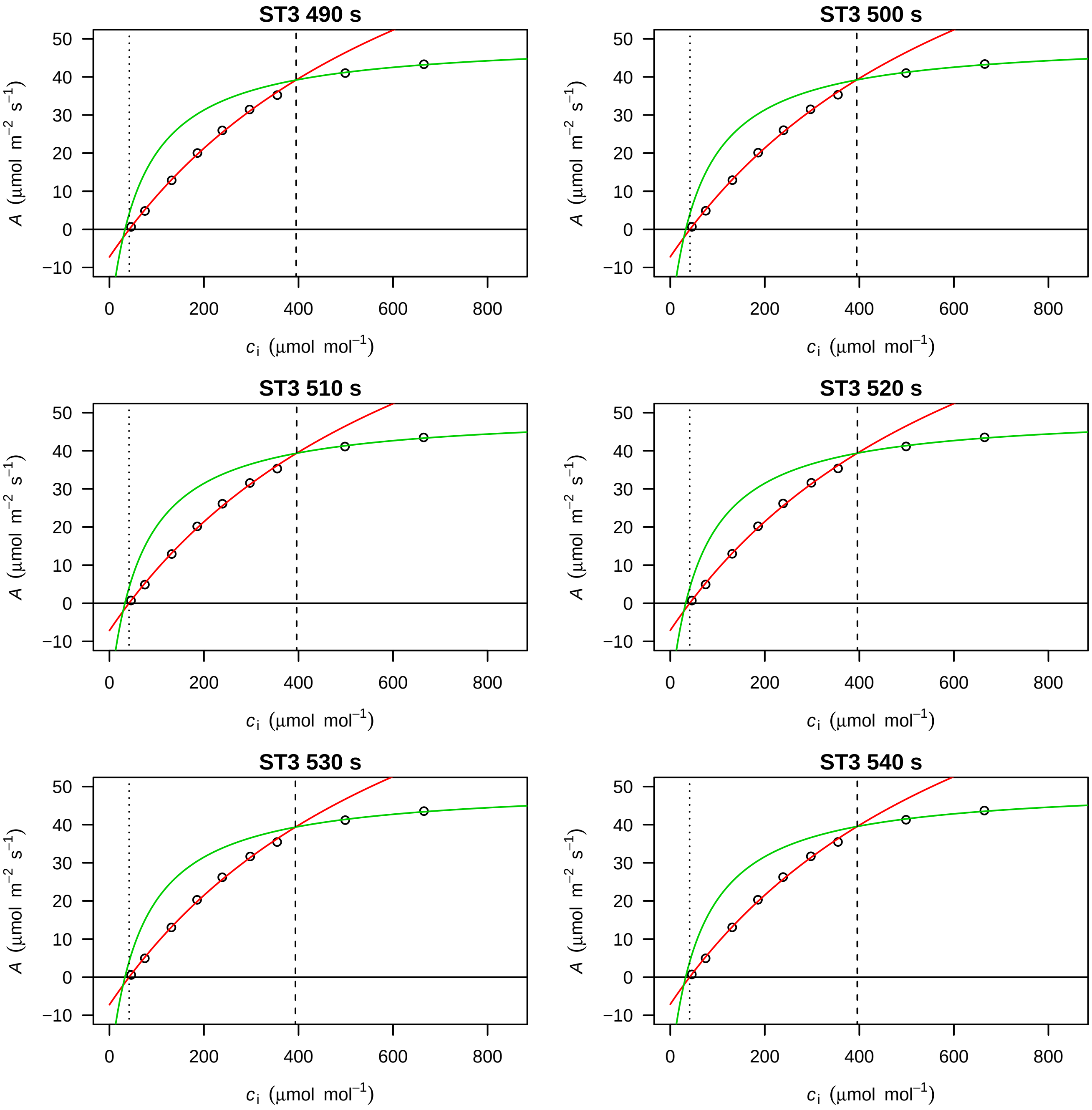
ST3 490 s
ST3 500 s
ST3 510 s
ST3 520 s
ST3 530 s
ST3 540 s
A (µmol m−2 s−1)
ci (µmol mol−1)
50
40
30
20
10
0
−10
0
200
400
600
800

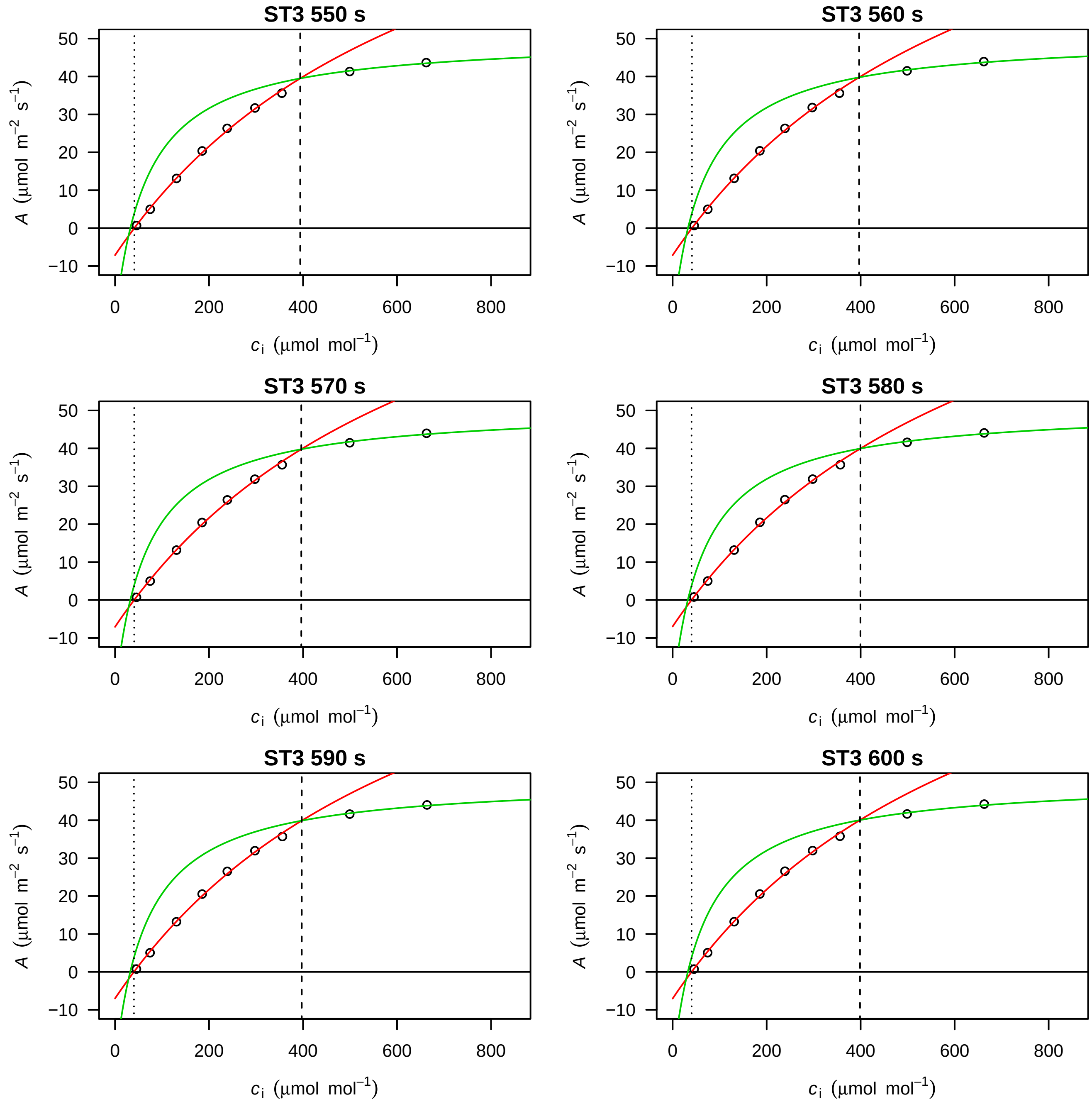

ST3 550 s
ST3 560 s
ST3 570 s
ST3 580 s
ST3 590 s
ST3 600 s
A (µmol m−2 s−1)
ci (µmol mol−1)
50
40
30
20
10
0
−10
0
200
400
600
800

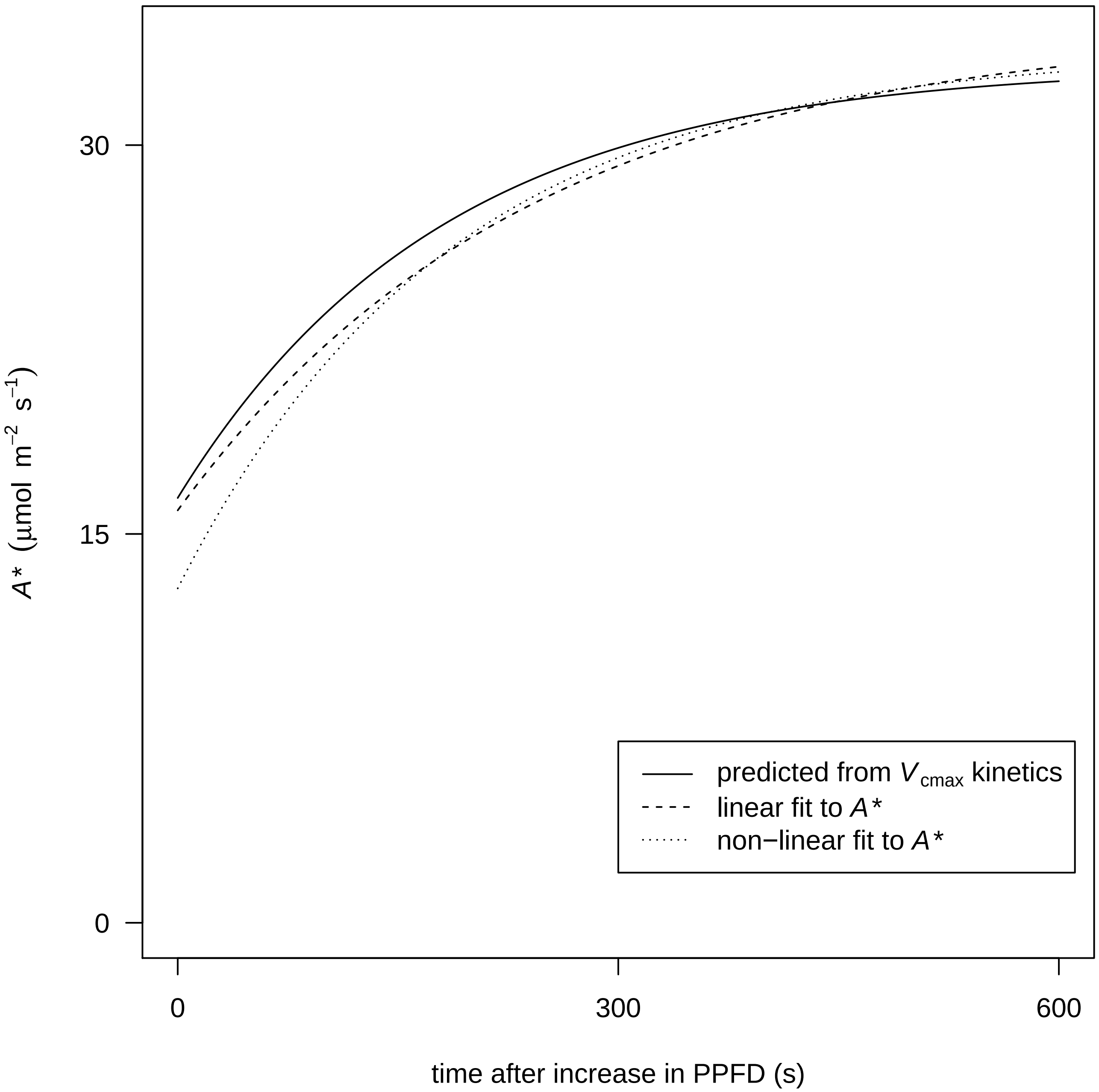
30
15
0
0
300
600
$A*$ (µmol m^{-2} s^{-1})
time after increase in PPFD (s)
predicted from V_{cmax} kinetics
linear fit to $A*$
non−linear fit to $A*$
